# Chemical Interactions and Their Role in the Microphase Separation of Block Copolymer Thin Films

**DOI:** 10.3390/ijms10093671

**Published:** 2009-08-25

**Authors:** Richard A. Farrell, Thomas G. Fitzgerald, Dipu Borah, Justin D. Holmes, Michael A. Morris

**Affiliations:** 1 Department of Chemistry, University College Cork, Cork, Ireland; E-Mails:r.farell@ucc.ie (R.A.F.);thomas.g.fitzgerald@intel.com (T.G.F.);d.borah@ucc.ie (D.B.);j.d.holmes@ucc.ie (J.D.H.); 2 Centre for Research on Adaptive Nanostructures and Nanodevices (CRANN), Trinity College Dublin, Dublin, Ireland; 3 Tyndall National Institute, The Maltings, Cork, Ireland; 4 Intel Ireland, Leixlip, Co. Kildare, Ireland

**Keywords:** block copolymer, intermolecular forces, self-assembly, ordering, structural regularity, thermodynamics

## Abstract

The thermodynamics of self-assembling systems are discussed in terms of the chemical interactions and the intermolecular forces between species. It is clear that there are both theoretical and practical limitations on the dimensions and the structural regularity of these systems. These considerations are made with reference to the microphase separation that occurs in block copolymer (BCP) systems. BCP systems self-assemble via a thermodynamic driven process where chemical dis-affinity between the blocks driving them part is balanced by a restorative force deriving from the chemical bond between the blocks. These systems are attracting much interest because of their possible role in nanoelectronic fabrication. This form of self-assembly can obtain highly regular nanopatterns in certain circumstances where the orientation and alignment of chemically distinct blocks can be guided through molecular interactions between the polymer and the surrounding interfaces. However, for this to be possible, great care must be taken to properly engineer the interactions between the surfaces and the polymer blocks. The optimum methods of structure directing are chemical pre-patterning (defining regions on the substrate of different chemistry) and graphoepitaxy (topographical alignment) but both centre on generating alignment through favourable chemical interactions. As in all self-assembling systems, the problems of defect formation must be considered and the origin of defects in these systems is explored. It is argued that in these nanostructures equilibrium defects are relatively few and largely originate from kinetic effects arising during film growth. Many defects also arise from the confinement of the systems when they are ‘directed’ by topography. The potential applications of these materials in electronics are discussed.

## 1. Introduction and Background

Self-assembly (and self-organisation) has become a topic of enormous interest as scientists have sought to provide chemical methodologies (sometimes called ‘bottom-up’ techniques) to create highly organised materials at scales beyond the crystallographic dimensions of atoms or simple molecules. These regular assemblies are expected to have applications as alternatives to photolithography (particularly when feature sizes are on the nanoscale) or where there might be advantages in high surface area or regular feature sizes. An example of the latter is the formation of regular sized pores for selective molecular separations, selective adsorption systems and high activity catalysis. Self-assembly is used as a term to describe spontaneous processes where nanoscale entities pack into regular arrangements in order to attain a minimum free energy through minimisation of repulsive and maximisation of attractive molecular interactions [1]. In self-assembly, defects can be considered as higher energy states whose concentration can, therefore, be low and this provides a thermodynamic driving force for the high structural regularity of the aggregate of entities [2]. In the simplest cases the self-assembling entities might be solid particles and Murray has pioneered the use of ligand chemistry to generate regular particulate aggregates as a means of enhancing or controlling the physical properties of the aggregate compared to individual particles [3]. Self-assembly might also be on the molecular scale involving surface molecules and described as self-assembled monolayers (SAMS) [4] or involving molecules in solution where the formation of vesicles or micelles provide suitable examples [5]. Perhaps the most successful and well known self-assembled nanostructured materials is mesoporous silica which has a number of important commercial applications including catalysis, material separations and sorbents [6]. The structures are so regular in these materials that the size monodispersed pore network can act as a template for the growth of regular sized nanoparticles and nanowires [7].

The term self-assembly has also been applied to processes not involving individual entities but also has been used to describe processes such as phase separation within a single component (as in the example described here, block copolymer microphase separation, and this is discussed in depth below). Phase separation can probably be more correctly described by the related term self-organisation. The difference between self-assembly and self-organisation can be difficult to differentiate [8]. Very often, in chemistry especially, self-assembly and self-organisation are used interchangeably and we will continue this practice in this article. Self-assembly is generally reserved for systems that are driven to equilibrium via physical interactions between entities (a free energy minimum). Self-organisation refers to a dynamic process where the assembled or organised structure is in a steady state. It is clear that in organised micelle systems where the lifetime of any individual micelle is relatively short that the system can be properly described as dynamic and the term self-organisation can be used appropriately. For BCP systems where a well-defined order-disorder transition is present the term self-organisation could be used but in many cases these systems reach a true equilibrium and the term self-assembly can be properly used. There is further confusion because many (but by no means all) BCP structures are micellar in nature. Assignment and proper description of these terms are beyond the scope of this article and for this reason the term self-assembly will be generally used as a generic term.

Self-assembly cannot be confused with chemical reactions; it is centred on arrangements of species mediated by weak, non-covalent, intermolecular forces. Self-organisation can often involve external mediating or directing forces as well as these weak intermolecular forces [8]. Fundamental to self-assembly is that a collection of randomly arranged components (or different parts of the same molecule) interact to form a more regularly arranged system. Note that the term order should be carefully avoided because this might imply that entropy always decreases in the self-assembly process. This is not so and in a few examples local entropy effects can outweigh decreasing entropy at larger scales. This can be the case in micelle formation and *e.g.*, in the case of colloidal self-assembly where the presence of secondary components (*i.e.*, smaller particles or polymers and inter-particle excluded volume) can increase entropy through the assembly process [9,10]. It should be noted that the micellar processes are true self-organisations because the assembly not only results from the intermolecular forces between the entities but also from interactions between the entities and the solvent [5]. Strong bonding covalent forces are not usually associated with self-assembly processes but these may be involved indirectly, most notably in the formation of self-assembled monolayers where a terminal or head group can provide a strongly bound ‘anchor’ to a substrate surface whilst tail groups interact via weaker intermolecular forces [11]. Self-assembly is often associated with the merging field of nanosciences but is a vital part of nature and is *e.g.*, responsible for the folding of nucleic acids into their functional forms [12].

Below, we examine in detail the microphase separation of block-copolymers describing the thermodynamics of the process, the type of structures formed, the formation of regular thin films and how the structures might be directed to define orientation and alignment, Applications of these systems are also discussed. As discussed above, BCPs can form ordered structures via self-organisation and self-assembly-although the generic terms self-assembly will be used through the rest of the article.

### 1.1. The Thermodynamics of Self-Assembly

Self-assembly is an equilibrium process that represents a balance between repulsive and attractive forces between entities [1]. These forces are manifest as a minimum in potential energy with distance apart and are discussed further below. This provides a useful framework for understanding and modelling the microphase separation of BCPs. The thermodynamics of the self-assembly process can be represented by a simple Gibbs Free Energy equation

(1) $${\Delta \text{G}}_{\text{SA}}={\Delta \text{H}}_{\text{SA}}-{\text{T}\Delta \text{S}}_{\text{SA}}$$

where self-assembly is a spontaneous process if ΔG_SA_ is negative. ΔH_SA_ is the enthalpy change of the process and is largely determined by the potential energy/intermolecular forces between the assembling entities. ΔS_SA_ is the change in entropy in the process. Since the organisation is generally (but not always) accompanied by an entropy decrease, for self-assembly to be spontaneous the enthalpy term must be negative and in excess of the entropy term. The equation shows that the self-assembly process will become progressively less likely as the magnitude of TΔS_SA_ approaches the magnitude of ΔH_SA_ and above a critical temperature will not occur.

**Figure 1.**
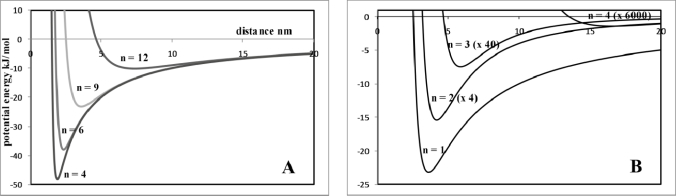
Potential energy against distance curves. A—result of increasing the short range nature of the repulsive forces between entities in a self-assembly process. Note the increasing width of potential energy well. B—result of increasing the short range nature of the attractive forces between entities in a self assembly process. Note the dramatic decrease in the depth of the well.

As regular structural arrangements are observed in self-assembly it is clear that there must be a balance of attractive and repulsive forces between entities or else an equilibrium distance would not exist between the particles. Since, for practical reasons, the assembly is generally not at the atomic or small molecule scale (for practical reasons outlined below) it is generally necessary that both attractive and repulsive forces are long range interactions (as distinct from short range chemical bonds) if the separation distance between features is to be in the nanometre range. This can be illustrated using very simple consideration of the intermolecular forces between the entities. If we assume that the attractive intermolecular forces can be modelled as an attractive potential between similar point charges (Q), the potential energy (V_att_) follows a 1/r dependence and can be written as

(2) $${\text{V}}_{\text{att}}=-{\text{Q}}^{2}/4{\pi ɛ}_{\text{o}}\text{r}$$

where r is the separation of the entities. The repulsive charge can be modelled as V_rep_ α 1/r^n^. Assuming a charge of around 1.37 × 10^−19^ C and a repulsive constant = 10000 (10^−9^)^n^ kJ mol^−1^ the variation in the total potential-distance curves (V_tot_ = V_att_ + V_rep_) as a function of n in the repulsion term can be plotted (Figure 1A). The curves describe a classic potential energy well with a minimum V_tot_ at an equilibrium separation distance between the entities. It is worth noting since RT ~ 2.5 kJ mol^−1^, that the minimum value of V_tot_ must be significantly greater than this to provide a driving force for assembly that compensates for an entropy decrease (Equation 1). The minimum value of V_tot_ can be approximately associated with ΔH_SA_ assuming that there is no volume or temperature change during self-assembly. The attractive term is long range in nature and the width of the potential energy well that is formed is defined, in this case, the repulsive term. At n=4 there is a well-defined potential energy minimum. This is important because it will precisely define an equilibrium distance between entities that is necessary if structural regularity is going to be high. The effect of increasing n, *i.e.*, increasing the short-range nature of the repulsive forces, is to reduce the value of the potential energy minimum, increase the width of the potential energy well and move the minimum to greater distances. The increasing shallowness of the well is a major problem in terms of generating patterns of high structural regularity because it ensures a variation in spacing between entities (or features in phase-separated systems outlined below) can exist with little energy cost.

The effect of increasing the dependence of potential energy with distance, *i.e.*, increasing the short-range nature of the attractive potential, also has a dramatic effect on the potential energy curve. This is modelled using a V_att_ that follows a 1/r^n^ dependence whilst using a constant repulsive term that varies as 1/r^6^. Illustrative data are shown in Figure 1B. Increasing the value of n reduces the value of the potential energy minimum, increases the width of the potential energy well and moves the minimum to greater distances as described for the repulsive forces above. However, as n increases the decrease in the value of the potential energy minimum is very considerable such that changing n from 1 to 3 reduces the potential energy minimum by a factor of around ~× 150.

Although these are quite simple calculations they do illustrate some important concepts in self-assembly on the mesoscale. Firstly, if the entities are to be relatively large distances apart, the repulsive and attractive forces between the entities will need to be relatively high or the potential energy will not provide an effective driving force at room temperature. Secondly, as the spacing between entities or features increases, variations in the separation distance within the self-assembled structure will increase dramatically and lead to poor structural regularity. Finally, for self-assembly to be effective, there needs to be a delicate balance of the intermolecular forces and because of this self-assembly with high structural regularity is not common-place and will require careful molecular or particle design coupled to optimisation of the process.

Finally, it should be stressed that self-assembly is a spontaneous chemical process where entities or components within a mixture arrange themselves in a structured manner and these processes take place in normal chemistry environments *e.g.*, solution mediated. Normally the self-organisation is borne from an initially disordered system. Importantly the equilibrium low-energy arrangement is reached from positional fluctuations as a result of thermal effects. Thus, the effective interaction potential between the entities or components can not exceed thermal energy by too great a factor or else it will not be possible to minimise positional errors in the in the arrangement. Alternatively, there has to be enough difference between thermal energy and the interaction potential energy to maintain order within the pattern. The thermodynamics of defect formation is described below (Figure 1).

### 1.2. The Intermolecular Forces Involved in Self-Assembly

A detailed understanding of the theory of self-assembly is a difficult problem and these difficulties have been discussed at length [13]. Very many kinds of intermolecular forces can be involved in self-assembly. These comprise the classic polar forces including ionic, ion-dipole, dipole-dipole and hydrogen-bonding and other variations that can be described as hydrophobic interactions. Π-π interactions and even weak covalent forces such as coordination forces have been associated with self-assembly. Π-π interactions have importance in block copolymer self-assembly because of the potential for stacking leading to supramolecular structures [14]. These latter forces may have particular relevance because they can accommodate classic inorganic chemistry to be applied to design systems [15].

Probably, the most important forces in self-assembly are often described as being hydrophobic in nature and are responsible *e.g.*, for protein folding and micelle formation of amphiphilic block copolymers in solution [16] and colloidal assembly. A hydrophobic force is essentially an entropic force but it does have an enthalpic component largely originating from dispersive forces between molecules [17]. In water, or similar solvents with strong H-bonding type interactions, the introduction of anon-hydrogen bonding surface (*i.e.*, aggregations of molecules principally interacting via dispersive and non-electrostatic forces) results in a rearrangement of the system minimise the number of disrupted hydrogen bonds and maximising the entropy of the system. These arrangements can lead to ordered assemblies of the hydrophobic components and is the basis of the so called “attraction” between hydrophobic objects in solution. It should be notes that the attractive force between the hydrophobic components is balanced by a weak repulsive force when they are close. This repulsion originates from the water molecules when trapped in very low entropy positions between the hydrophobic components. In this way, London dispersion or van der Waals forces are important hydrophobic interactions. Although these are normally associated with being relatively weak in molecular systems, they become stronger as size and surface area increase.

On the basis of even this very brief review of the role of intermolecular forces in self-assembly, it is, therefore, not surprising that self-assembly, self-organisation and phase separation are highly sensitive to environment and substrate effects [18]. It is beyond the scope of this article to explore these forces in greater details. Instead we will concentrate on the effects of these forces on the self-organisation or microphase separation of block copolymers and in particular the understanding of defect formation in these systems.

## 2. Microphase Separation and Self-Organisation Block Copolymers

Block copolymers have become increasingly more important materials as routine design and synthesis of these materials has become practical. Block copolymers were developed to essentially tune the properties of the macromolecule between that of the two blocks individually. The advantage of using a single macromolecule rather than a blend is that the macroscopic phase separation in mixtures can not occur. However, the chemical mismatch does lead to microphase separation as described below. Industrial synthesis of block copolymers (BCPs) was first demonstrated in the 1950s by scientists at BASF and ICI around the generation of triblock systems of poly(ethylene oxide) and poly(propylene oxide). Amongst many applications these found widespread use as surfactants, anti-foaming agents, cosmetic materials and drug release materials [19]. More recently they have found use as versatile ‘templating agents’ for the generation of ordered nanoporous silicates allowing precise control of pore diameters [20]. Spandex was the first BCP to be widely known because of its use in textiles (spandex is an anagram of expands) and was invented by the DuPont chemist J. Shivers [21]. It became apparent that the possibility of forming macromolecules with blocks of differing chemical properties could yield materials where the interaction of the different blocks would ordain important physical properties. Many aspects of BCPs have been reviewed in depth [22,23]. This article will be restricted to discussion of the formation of nanopatterns of these materials in thin film form on substrates. The nanopatterns are essentially the result of the self-organisation *via* microphase separation of the BCP at the surface and not *via* micelle formation and related phenomena of the BCP in solution. Lyotropic phases will not be discussed at length here, however, solvent effects can not be completely ignored because it is convenient and practical (particularly for the thin films discussed here) that the polymers are solvent cast onto the substrate surface by techniques such as dip- and spin-coating. Further, a technique known as solvent-annealing or solvent-swelling is becoming common place as a means of attaining high degrees of structural regularity. This ordering is a result of the increased mobility within the macromolecule block network related to the decrease in the glass transition temperature as a result of solvent molecule inclusion [24].

### 2.1. Intermolecular Forces in Microphase Separation of Block Copolymers

When any two polymers are mixed the result is often phase separation. This phase separation may not be observed on a macroscopic scale; unlike phase separation in liquids, the process may be extremely slow because of the mass transport limitations associated with the large number of mers in the polymer. If ordered systems are observed this process may be properly described as self-organisation. Very often phase separation in a polymer blend will not be observed until heating to around the glass temperature when chain mobility is much higher. It is worth noting that even when the polymers are quite similar chemically, small differences can result in strong repulsive interactions between the polymers because of the number of units in a chain. The polymer molecular weight will play a pivotal role in any phase or microphase separation process because it will define both the strength of the repulsions and chain mobility within the system. The repulsive forces between blocks will lead to segregation of two polymer components under suitable temperature conditions. Importantly, there is a practical temperature window such that the temperature should be i) low enough that the result of intermolecular forces can be expressed despite thermal randomisation and ii) high enough that phase separation can be achieved in reasonable times.

In block copolymers complete phase separation of the chemically distinct sub-groups can not be achieved because of the chemical bonds that bind the two blocks. Thus, the chemical immiscibility of the monomers that would drive a blend of polymers to segregate is counter-balanced by a restorative entropy cost associated with deformation of the random coil structures of the blocks that occurs during microphase separation. The result of this balance of repulsive intermolecular forces between blocks and attractive restoring force is the formation of mesocale regular periodic structures of microphase separated domains with the structure being formed in order to minimise the contact area between dissimilar blocks. The term microphase separation is becoming strongly associated with this BCP self-organisation but can also be seen in mixtures of liquids, metal alloys and ceramic systems.

The thermodynamics of microphase separation can be expressed by modification of Equation 1

(3) $${\text{G}}_{\text{mix}}-{\text{G}}_{\text{PS}}={\Delta \text{G}}_{\text{SA}}={\Delta \text{H}}_{\text{SA}}-{\text{T}\Delta \text{S}}_{\text{SA}}$$

where G_mix_ and G_PS_ represent the free energy of the mixed and phase separated systems respectively. ΔH_SA_ and ΔS_SA_ represent the enthalpy/entropy changes between the mixed and phase separated states respectively. It should be noted that the entropy change associated with mixing is usually quite small because of the random coil confirmation adopted by the polymer blocks. Within the mixed state the BCP will adopt an unsystematic confirmation of random coils which results in a great many contacts between the different blocks. The random coil arrangement is the usual structure for macromolecules because of the possible rotations around the C-C bonds in the polymer backbone. The intermolecular forces driving the microphase separation process are normally of the hydrophobic type described above where the attraction of more polar components forces the non-polar components to aggregate. This segregation of the blocks reduces the number of interactions of the dissimilar blocks thereby lowering the number of repulsive interactions between chains. The value of ΔH_SA_ is thus always negative and should result in de-mixing of the blocks. During segregation of the blocks the random coils become extended decreasing the number of possible configurations and so decreasing entropy. This results in an entropic restorative force opposing the enthalpy driven phase separation and so phase separation occurs over very limited distances of the same order as the random coil length.

The intermolecular forces dominant in microphase separation are dispersion forces, polar forces and hydrogen bonding. These give rise to the balance of repulsive and attractive interactions (‘hydrophobic forces’) detailed above. The application of the models to explain BCP self-assembly has significant relevance and has even been used to explain the self-assembly associated with Huntington’s disease [25]. Dispersion forces become dominant in polymers that contain hydrocarbon groups and although relatively weak can be quite strong over the volume of the macromolecule. Polar forces are common in oxygen containing systems such as polyesters and polyethylene oxides. Hydrogen bonding is common in many systems including polyamides. The strength of the intermolecular forces can be understood by consideration of a term known as the solubility parameter which was first proposed by Hildebrand [26] and has been extensively reviewed because of its’ fundamental importance [27–28]. Although this term is often considered to be a qualitative measure of the propensity of one material to dissolve in another, the solubility parameter, δ, is a thermodynamic quantity quite specifically defined. The cohesive energy E_coh_ of a substance in a condensed state is defined as the increase in internal energy U per mole of substance if all of the intermolecular forces with its environment are eliminated. In a solution the free energy change represents the difference between the condensed (i) and the gas (g) phase states (ΔU = U_g_ - U_i_). Since the internal energy (ΔU) of a system is the sum of kinetic energy and potential energy it can be seen that E_coh_ is closely related to the strength of the intermolecular forces between the entities in the condensed state but the kinetic energy component can not be ignored because significant differences are expected. The E_coh_ can be expressed as the cohesive energy (molar volume) density (ECD)

(4) $$\text{ECD}={\text{E}}_{\text{coh}}/{\text{V}}_{\text{m}}$$

and the solubility parameter, δ, is defined as

(5) $${\text{ECD}}^{1/2}=\delta$$

And

(6) $$\delta ={\left({\text{E}}_{\text{coh}}/{\text{V}}_{\text{m}}\right)}^{1/2}$$

(7) $$\text{Since}\;\Delta \text{U}=\Delta \text{H}+\Delta (\text{P}\;{\text{V}}_{\text{m}})$$

*i.e.*, the internal energy change is equal to the change in PV work and the enthalpy change we can write

(8) $$\delta ={\left(({\Delta \text{H}}_{\text{vap}}+\text{RT})/{\text{V}}_{\text{m}}\right)}^{1/2}$$

where ΔH_vap_ is the heat of vaporisation. The solubility parameter should be referred to as the cohesion parameter when discussing non-liquid materials. The relationship of δ to the intermolecular forces can be seen using Hansen parameters developed in 1966 [30,31]. Hansen parameters separate the Hildebrand value into three parts: a dispersion force component, a hydrogen bonding component and a polar component. The square of the Hansen parameters are additive

(9) $$\delta^{2}=\delta_{\text{d}}^{2}+\delta_{\text{p}}^{2}+\delta_{\text{h}}^{2}$$

where δ_d_ is the dispersion component, δ_p_ is the polar component and δ_h_ is the hydrogen bonding component. Numerical values for the component parameters are estimated by reference to the non-polar molecule that most closely resembles the molecule of interest (*e.g.*, polyethylene for polyvinyl chloride). The contribution of polar and hydrogen bonding terms was based simply on other supporting evidence. Typical Hansen parameters are provided elsewhere [29,30].

The power of the solubility parameters and, in particular, Hansen parameters is that they can be used to predict the solubility of one component in another particularly through consideration of δ_A_^2^ – δ_B_^2^. If this is close to zero it implies that the molar enthalpies of vaporisation are similar and the intermolecular forces holding the components in a fluid or solid state similar. For example, if we take two polymers such that δ_A_^2^ – δ_B_^2^ has a significant magnitude then it would be expected that they would phase separate on mixing. If the components are present as a block copolymer they would be expected to microphase separate. The Hansen approach suggests that raw Hildebrand values may not provide adequate understanding in all systems since we would hope that polar and non-polar contributions of the two components should also be similar for favourable mixing or lese that segregation of components would be expected on the basis of the hydrophobic forces described above. It should also be noted that solubility or mixing of polymer components will be very sensitive to even small differences in the solubility parameter of the components. This is due the square dependence and also because it is a direct measure of the magnitude of the intermolecular force differences and even when they are small can be very significant over the whole of the macromolecule. This is described and illustrated in detail below.

### 2.2. The Solubility Parameter and the Thermodynamics of Microphase Separation

The thermodynamics of microphase separation in BCPs has been reviewed several times following the original work of Bates [31]. The theory will not be detailed in depth here except to show how it relates to intermolecular forces through the solubility parameter. Most of the understanding of microphase separation of BCPs is centred on a term known as the interaction parameter χ. Assuming a simple diblock copolymer made up of sub-units A and B, the χ value resulting from the interactions between block A and block B can be written as

(10) χ=zΔw/kT

and χ is the exchange energy per molecule normalised by the thermal energy kT and is dimensionless. The number of neighbours surrounding one block is z. Δw is the exchange energy which is the difference in energy between the interaction between block A and block B and the average of the self interactions between block A-block A and block B-block B. That is, Δw is the energy cost of taking a block of A from surrounding A blocks and placing in a B block environment and doing the same for a B block (from a B environment to an A environment). The interaction parameter can be related directly to the molar enthalpy change of mixing, ΔH_m_, by

(11) $${\Delta \text{H}}_{\text{m}}=f_{\text{A}}f_{\text{B}}\;\chi \text{RT}$$

where ƒ_A_ and ƒ_B_ are the volume fractions of the blocks. By conventional solution theory and assuming no volume change on mixing, it can be shown that

(12) $$\chi ={\text{V}}_{\text{m}}{\left(\delta_{\text{A}}-\delta_{\text{B}}\right)}^{2}/\text{RT}$$

where δ_A_ and δ_B_ are the solvent parameters (see below) of the two blocks. Therefore

(13) $${\Delta \text{H}}_{\text{m}}=f_{\text{A}}f_{\text{B}}\;{\text{V}}_{\text{m}}{\left(\delta_{\text{A}}-\delta_{\text{B}}\right)}^{2}$$

This is important because it shows that any block copolymer system where the blocks have different solubility parameters (*i.e.*, different strengths and forms of intermolecular interactions) will have a positive enthalpy of mixing and will, thus, have a tendency to microphase separate and self-assemble provided the entropy change (always a decrease as discussed above) associated with the process is not too large as to overcome the enthalpy contribution. Flory-Huggins theory has been the basis for modelling the behaviour of block copolymers since their invention and remains the most used model to date [32,33] providing a robust basis for the prediction of morphology seen in BCP microphase separated systems. Using this formulism the configurational entropy of phase separation is assumed as the only major contribution to energy such that the entropy associated with microphase separation ΔS_m_ = klnΩ where Ω is the number of possible ways of arranging the system. *Via* Stirling’s approximation the entropy change can be written as can be written as

(14) $${\Delta \text{S}}_{\text{m}}/\text{RT}=(1/{\text{N}}_{\text{A}})\text{ln}\;f_{\text{A}}+(1/{\text{N}}_{\text{B}})\text{ln}\;f_{\text{B}}$$

where N_A_, N_B_ are the degrees of polymerisation of each block such that ƒ_A_ = N_A_/(N_A_ + N_B_). Since the entropy decreases in the system on mixing and using Equation 11

(15) $${\Delta \text{G}}_{\text{m}}/\text{RT}=f_{\text{A}}f_{\text{B}}\;\chi +(1/{\text{N}}_{\text{A}})\text{ln}\;f_{\text{A}}+(1/{\text{N}}_{\text{B}})\text{ln}\;f_{\text{B}}$$

**Figure 2.**
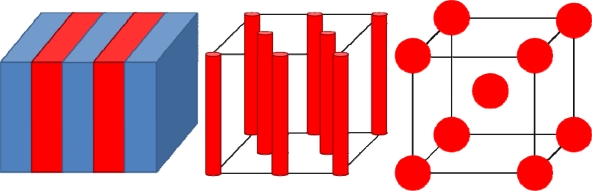
Schematic showing the most useful microphase separated arrangement of blocks possible in a diblock copolymer as a function of increasing compositional asymmetry. The gyroid structure is not shown (see text).

This Equation specifically relates to the mixing process and not phase separation. The implication is that the free energy of mixing is always likely to be positive bearing in mind the definition of χ given in Equations 10 and 12. The driving force for self-assembly is the minimisation of the free energy of mixing by the regular patterns formed by microphase separation. For illustrative purposes consider the formation of a regular, microphase separated, lamellar phase consisting of alternating stripes of blocks from a AB block copolymer with ƒ_A_ = ƒ_B_. The lamellar structure is a common motif in phase separation because it is achieved with lowest mass transport limitations. This is particularly important considering that phase separation is limited by the covalent bonding between blocks and all theories suggest this is the lowest energy structure for BCPs. A simple schematic of the arrangement can be seen in Figure 2. As can be seen from Equation 3, ordered self-assembly/microphase separation will occur provided that ΔG_SA_ = G_mix_ – G_PS_ is negative. The free energy change in forming the lamellar structure (ΔG_SA,L_) can be described by modelling G_mix_ as a sum of AB contacts and G_PS_ as a Hookian term describing the balance of repulsive enthalpic and attractive/restorative entropic forces (as detailed above) plus an interfacial term. In this way [31,34] the G_PS_ can be written

(16) $${\text{G}}_{\text{PS}}=1.19{\left(\chi_{\text{AB}}\text{N}\right)}^{1/3}$$

and the equilibrium spacing between stripes in the lamellar structure (L) as

(17) $$\text{L}=1.03\text{a}{\left(\chi \text{AB}\right)}^{1/6}{\text{N}}^{2/3}$$

Since the sum of simple contacts in the mixed system allows G_mix_ to be estimated as (χ_AB_N)/4 it is possible to write that for microphase separation to occur G_mix_ must be greater or equal to G_PS_ and the minimum condition is

(18) $$1.19{\left(\chi_{\text{AB}}\text{N}\right)}^{1/3}=(\chi_{\text{AB}}\text{N})/4$$

Thus, for microphase separation χ_AB_N must be greater than 10.4. Since χ_AB_ is a measure of the chemical dis-similarity between the units (mers) in the blocks χ_AB_N represents the total dis-similarity over the whole macromolecule. Using Equation 18 the minimum value of (χ_AB_N)_min_ to bring about phase separation is about 10.4. This very simple approach provides a value for (χ_AB_N)_min_ which is very similar to much more complex theories developed by Leibler using self-consistent field theory [35]. A summary of recent theoretical developments in block copolymer phase separation has been provided by Grason [36].

### 2.3. Phase Diagrams

The structure resulting from microphase separation will be dependent only on a limited number of parameters; the chemical differences between blocks, the degree of polymerisation and the composition of the BCP. Since the interaction parameter is dependent on temperature, this will also have an effect. The variation of structure with χ_AB_N and composition is normally shown using a phase diagram and was first theoretically determined by Matsen [34] and is still very widely used to describe self-assembly in these systems. Only simple diblock systems will be described in detail here and the reader is referred to phase diagrams for triblock structures [31]. The general form of the theoretical phase diagram calculated by Matesen agrees well with literature [37]. The phase diagram is separated into several regions which are characterised by the relative stability of the structures formed and the dependency of the separation of features or feature size, L, on the degree of polymerisation [38]. For the symmetric system (*i.e.*, ƒ_A_ = ƒ_B_) below χ_AB_N ~ 10.5 the system is disordered. Between χ_AB_N = 10.5 and 12.5 the region is known as the weak segregation limit and L is proportional to N^0.5^. In the intermediate regime, χ_AB_N = 12.5 to 95, L is proportional to N^0.72^ and above this level (the strong segregation limit) L is proportional to N^0.67^. For practical purposes, it is probably best to avoid the weak segregation limit for creating well-defined block copolymer structures, because although this gives the smallest feature size, the thermodynamic driving force (as determined by the intermolecular forces) is small and regions of disorder might be expected because of thermal effects. The strong segregation limit may also afford problems in the commercial use of these materials because the thermodynamic driving force for microphase separation is becoming so strong that it vastly exceeds thermal energy and achieving thermal equilibrium (for reduction of disorder) may require extended annealing. For practical purposes it should also be remembered that as the molecule increases in length there will be increased rigidity and the glass transition temperature of the systems will increase and there will be temperature requirements imposed by processing in commercial applications which might place an upper limit on the time used to attain phase separation.

In the region of the diblock phase diagram where the balance of intermolecular forces results in microphase separation various structures form within certain composition limits. The phase diagram first described by Matsen [34] is symmetrical around the mid point of composition ƒ_A_ = ƒ_B_ = 0.5 although experimentally the system is a little more complex than predicted [39]. This central region adopts a lamellar structure. As ƒ_A_ increases to between 0.6 and 0.7 a bicontinuous gyroid phase of cubic symmetry consisting of interpenetrating tubules of the B block in A block is formed. This phase has a relatively narrow stable composition range particularly in the intermediate and strong segregation ranges. At higher ƒ_A_ values there is a composition range where there is a hexagonal arrangement of block B cylinders in a matrix of A. Finally, the B block adopts an arrangement of body-centred spheres. Due to its structural complexity and a narrow stability range, the gyroid structure has only limited applicability and is less well studied. The lamellar and, hexagonal and body-centred structures have been intensively studied and these are described in Figure 2. Of these the lamellar and hexagonal structures are most important in terms of patterning electronic materials and generating useful porous materials.

In reality, more complex structures do form because of the complexity of the intermolecular interac tions in polymers. Grayson has reviewed extensions to theory which allow more complex systems to be examined [40]. A structure of this type that has received some attention is the hexagonal-perforated-lamellar structure that is metastable and observed at the phase boundary between the lamellar and cylinder arrangements [41,42]. This perforated phase has become more important in thin film structures because it can be stabilised by surface reconstruction [43] due to preferential interactions of one block with gas, liquid or substrate interfaces. Hamley has surveyed a great number of diblock systems and the phases that they form [44].

### 2.4. Applications of Microphase Separation—SURFACE Pattern Formations

Microphase separation in BCPs is becoming a subject of research for potential commercial development. Few self-assembling systems can rival the regularity of BCP systems with perhaps only mesoporous silicates [45] and porous anodic alumina [46] rivalling the polymer systems in the feature size range of interest and having been shown to offer opportunities for controlled alignment. It can be seen from the arguments made above that the high regularity of the systems is because the intermolecular forces that drive the self-assembly are such that highly periodic structures are favoured and ordering can be attained at practical temperatures. One of the advantages of BCP self-assembly compared to these inorganic systems is that the film can be ‘annealed’ after their formation to improve the regularity of the self-assembled structures. Other self-assembled systems such as nanoparticle superlattices [47] also produce highly regular and sometimes complex structures. However, the synthesis of size mono- dispersed particles is challenging for all but a few systems and thin films of these tend to lack thermal and mechanical robustness. BCP systems do have an unrivalled combination of advantages; thin films can be formed from simple solutions, the resultant films are robust, the feature size is highly controllable using polymer engineering and the films are readily processed (*e.g.*, in pattern transfer where the polymer pattern is transferred to the surface by selective etch processes).

Authors have demonstrated many applications for microphase separated BCP thin films. BCP micelle systems have found commercial use in applications such as drug delivery but these are not the focus of the work described here and the reader is directed to some excellent reviews [48,49]. Applications for BCP films in the general area of materials science include solid state battery electrolytes [50] and membrane separation technologies [51]. Park and co-workers have provided an extensive review of technologies that might be developed using BCP thin films and these are largely in the area of development of strategies to develop nanoscale electronics, magnetics and photonics [52]. These ICT focussed technologies include low dielectric materials for electrical insulation and reduction of crosstalk [53–55], high density magnetic storage media [56] and photonic band gap crystals [57].

By far the most researched area for use of these materials is as potential alternatives to conventional mask-based photolithography for fabrication of nanoelectronic circuitry. Photo-lithography has been the cornerstone of the electronics industry since the advent of the first silicon devices [58,59]. The photolithographic process has been continually developed to allow the size of devices to be decreased and the density of devices constantly increased so that individual transistor sizes have shrank from cm type sizes to around 50 nm. The trend in resolution enhancement was, for many years, achieved by reducing the dimensions of the mask patterns whilst simultaneously decreasing the wavelength of the radiation (light) [60]. Currently, techniques such as immersion technologies whereby a liquid (usually water) is placed directly between the final lens and photoresist surface resulting in a resolution enhancement defined by the refractive index of the liquid have allowed device engineers to pattern transfer feature sizes (65 or 45 nm generation) that are actually less than the wavelength of light used (193 nm) [60]. Device performance is ultimately limited by the density of transistors on the chip [61] and it is clear that patterning requirements will continue to the 32 nm node and beyond. Although photolithography can potentially be used to create sub-10 nm device structures for high volume manufacturing processes, it will necessitate the use of deep UV (13 nm) and x-ray sources and these are associated with high costs and materials implications for the masks and resists [62].

For these reasons, self-assembly may have importance for transistor manufacture beyond the 22 nm node [63]. The advantages of self-assembly over conventional and non-lithographic methods include: (i) the reduction of source costs, (ii) elimination of masks and photoresists, (iii) non-existence of proximity affects, (iv) the possibility of developing 3D patterning techniques, (v) absence of diffraction restrictions to resolution and (vi) can be used to pattern materials with precision placement techniques by availing of templating (*i.e.*, deposition of materials within the structure, known as graphoepitaxy) or a chemical pattern (alternating surface chemistries). The microphase separation of block copolymers is emerging as the most promising method of assembling highly ordered nanopatterns at dimensionalities and regularity approaching the future device dimension requirements. These requirements are extremely challenging for self-assembly and lithography alike and include sub-nm line edge roughness and sub-4 nm positioning (of a feature expressed from the overlay registry requirements) accuracy for the 16 nm technology node [64]. The potential application of BCPs in this area has been extensively reported and reviewed [58,59,65–71]. These reviews also detail the methods by which the polymer nanopatterns can be processed into active components (*i.e.*, nanowires, nanodots of semiconducting, magnetic or conducting materials). As briefly outlined above, these are conveniently divided into two broad areas; pattern transfer where essentially the polymer is used as a mask within an etch process and templating where active materials are selectively placed at one block (or the space created by removal of one block). In the remainder of this article we will explore some of the issues which may prevent implementation of BCP self-assembly as a technology for development of nanoelectronic circuitry. The issues mainly result for difficulties in controlling or defining the chemical interactions of the polymer with the substrate and other interfaces.

## 3. Surface, Interface and Related Effects

The simple theories mentioned above for describing microphase separation relate to the development of bulk structures from the melt. However, for application in electronics the only practical means of developing thin films (<100 nm) is deposition from a solvent and, further, once formed the film is susceptible to boundary, thickness and interface effects which will disturb the delicate balance of intermolecular forces within the films.

### 3.1. Surface Interface Effects

Within mobile systems such as BCPS which are heated to around their glass transition temperature to effect microphase separation, changes in the film structure will occur to minimise surface energies and maximise bonding interactions with the surface. In block copolymer systems these interactions are manifest as: 1) preferential wetting layers by segregation of the preferred block to the substrate or surface, 2) irregular films caused by de-wetting of the film and 3) orientational effects and stability of unexpected film patterns.

**Figure 3.**
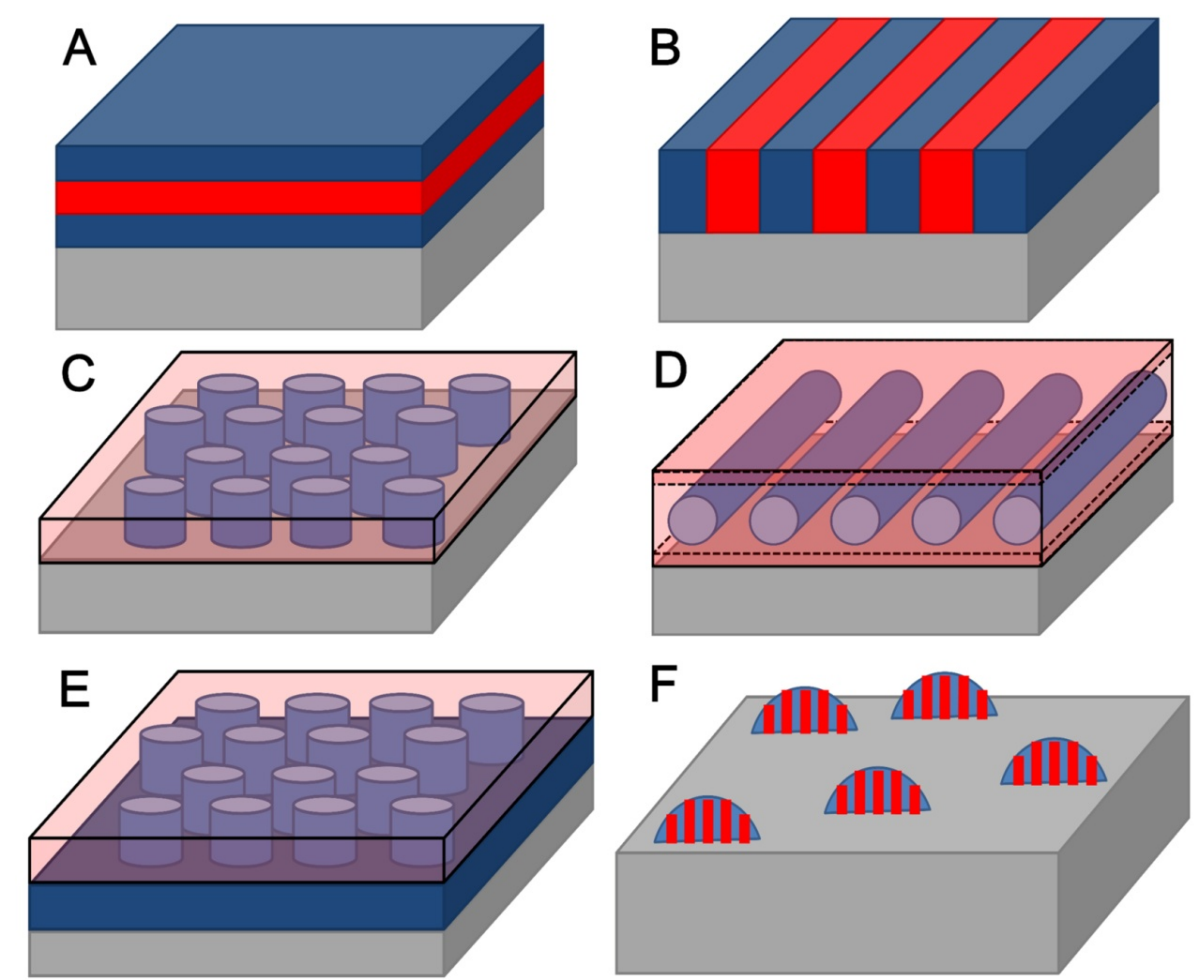
Orientational effects in block copolymer thin films upon self-assembly. Dashed lines indicate the wetting layers. See text for details.

Some examples are shown in Figure 3 (assuming substrate surface effects are dominant and surface interface effects can be ignored for simplicity). In Figure 3A and 3B two orientations of the same structure can be seen. In Figure 3A a horizontal orientation of the lamellae occurs if one block is preferentially favoured at the substrate surface whilst in Figure 3B a vertical orientation occurs as of both blocks are equally favoured. Similarly for the hexagonal cylinder structure, a substrate surface that interacts equally with both blocks will favour vertical orientation of cylinders with respect to the surface (Figure 3C). Whilst favourable interactions of one block with the surface and substrate interfaces can lead to the formation of wetting layers which will tend to favour a horizontal orientation to the surface (Figure 3D). In general the block that has lowest surface energy will reside at the surface. The substrate interactions can also yield complex structures. In Figure 3E strong substrate interactions with the minority block can lead to a perforated lamellar-like structure. The perforated lamellar structure is only pseudostable and is best thought of as an intermediate phase between the hexagonal and lamellar structures [72]. This structure is being observed more frequently in very thin films largely because of these strong interface effects [73]. The example in Figure 3E might arise if the substrate favours one of the blocks and the surface energies of both blocks are similar.

**Figure 4.**
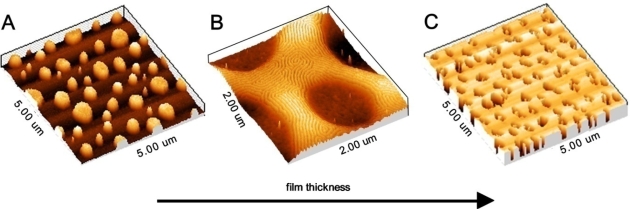
Phase separation of a PS-*b*-PMMA (symmetric, lamellar forming polymer) spin-coated thin film as indicated by tapping mode atomic force microscopy. The molecular weight of both the PS and PMMA blocks is 37 k. As the amount of polymer increases the morphology shows three distinct forms.

Finally, it should be recognised that the interactions of the BCP with the substrate can lead to complex morphologies on the micron scale. For example, if a surface is hydrophobic and both blocks in a diblock copolymer are hydrophilic there will be a tendency to form droplets rather than commensurate films as illustrated in Figure 3F where hemispheres of phase separated polymers are formed [74]. In the case of block copolymers commensurate (flat films of regular thickness) thin films are defined by the amount of polymer at the surface so that an integer (if the same block resides at the substrate and the surface interfaces) or half integer (*i.e.*, different blocks at the substrate and the surface interfaces) number of lamellae are formed [75]. The types of pattern produced are shown in Figures 4A–4C for a polystyrene-*b*-poly(methyl methacrylate) (PS-*b*-PMMA) BCP.

At lower thicknesses isolated islands of BCP are formed but as the amount of polymer increases these bumps merge and a bicontinuous layer is formed. As an amount of polymer equivalent to a commensurate layer is approached the film adopts a uniform thickness with a series of holes. Microphase separation is possible in these structures as shown in Figure 4. It should be noted that these images are relatively simple. Film thickness plays an important role in determining orientational effects—*e.g.*, in hexagonal cylinder arrangements whether the cylinders are vertical or parallel to the surface. In thin films the elastic strain energy associated with forming a parallel arrangement is too great unless an integer number of layers of cylinders are formed and instead a vertical arrangement is favoured [76]. In thicker films the strain energy can be more readily accommodated and thickness dependence is less. In general, very thin films favour vertical (to the surface) alignment of cylinders or lamellae. This is a challenge for lithographic applications because using the BCP nanopattern to create wire type structures (from cylinder forming structures) is significantly more difficult than forming nanodot structures. Because of this dependence of orientation with thickness, complex morphologies such as hemispheres can show several different orientations within a single object [77].

**Figure 5.**
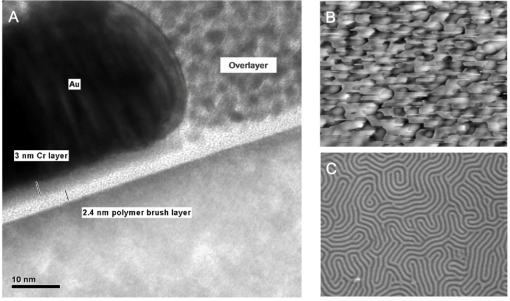
(A) cross-sectional TEM image of a random copolymer brush layer applied to a silicon substrate. The overlayer is a PS-*b*-PMMA BCP applied above it. The Au and Cr shown form pattern for graphoepitaxial alignment (see below). (B) a tapping mode AFM image of a lamellar forming PS-*b*-PMMA system on bare silicon whilst (C) is the same polymer on a well-defined brush layer.

These substrate effects need to be carefully controlled for applications in lithography where orientational control, alignment control and film regularity are pre-requisites for transferring patterns over large areas. Note here that orientation is used to describe direction of the BCP features relative to the surface plane (*i.e.*, vertical or horizontal) whilst alignment refers to determination of direction within the surface plane (to an azimuthal direction). The most obvious method of providing ideal or neutral surfaces is to manipulated the chemical interactions between the BCP and the substrate such that they do not differentiate between the blocks. Random block copolymer thin films (*i.e.*, where relatively short chains of each block provide a random distribution of composition) or self-assembled monolayers (SAMS) have provided a useful approach for this surface chemical modification. SAM methods have produced interesting results and orientational control has been demonstrated [78]. One of the more attractive features of the SAM chemistry is that for optimisation of BCP film quality it is necessary to achieve exact monolayer coverages of the SAM or polymer brush and this is readily achieved in SAMS by relatively simple methods. Polymer brush chemistry, largely developed by Russell, Hawker and their co-workers [79], also provides almost ideally chemically tailored surfaces for BCP self-assembly but obtaining very uniform surface coatings is more problematical than for SAMs, a typical example of a brush layer is shown in Figure 5. The importance of good brush chemistry for generating ‘perfect’ self-assembly has been shown by Nealey [80] and is demonstrated in Figure 5. Here a symmetric PS-*b*-PMMA block copolymer has been deposited onto a bare (100) silicon substrate and a brush modified version of the same surface. It can be seen that the unmodified surface, Figure 5B, produces little regular assembly because of poor polymer wetting characteristics. However, in the brush modified surface, Figure 5C, discrete lines are observed due to vertical orientation of the lamellar. These surfaces are often described as neutral—the term being used to indicate a surface wet by both blocks equally.

### 3.2. Solvent Interface Effects

The second interface of importance in polymer thin film formation is frequently a polymer thin film–solvent interface. For convenience polymer thin films are formed by conventional coating techniques (such as spin-coating or dip-coating) from polymer solutions. During solvent annealing/swelling the process of solution is complex. In the first part of the process solvent penetrates the macromolecular network forcing the individual molecules apart. This process results in an expansion in volume of the polymer and in thin films is associated with a thickness change which can often be revealed as a distinct colour change. This is known as polymer swelling. In high solvent concentrations the limited availability of polymer results in eventual complete separation of the chains and solution occurs. Where the amount of solvent is limited (*e.g.*, in solvent atmospheres or limited solubility), complete solution will not occur. The basis of polymer swelling, solution [81] and kinetics [82] has been described. In microphase separated BCP systems the chemical interactions between the polymer and the solvent can usually be ignored. This is because the system is usually heated at temperatures around the glass transition temperature but below the order-disorder temperature in vacuum to obtain the most ideal ordering possible and thereby minimising the total free energy of the system. In these treatments it is generally thought that solvent within the matrix will be rapidly removed compared to usual heating times. This may not be true for glassy polymers such as polystyrene heated well below the glass transition temperature. Recent work has shown that solvent may be retained in thin polystyrene films for extended periods [83]. Even in cases where polymer is removed by post-processing, interactions of the polymer may have a profound effect because of two effects. Firstly, the BCP with two distinct chemical blocks may form in solution. These micelles may have enough rigidity or mass transport limitations such that they survive and heating of the resultant thin film and examples are given below. Secondly, a technique known as solvent annealing is attaining popularity for bringing about microphase separation as an alternative to or in combination with vacuum heating.

Solvent annealing as an alternative to thermal annealing was proposed by Libera *et al*. [23] where microphase separation of a cylinder forming polystyrene-*b*-polybutadiene-*b*-polystyrene (PS-*b*-PB-*b*-PS) triblock copolymer was achieved using a solvent exposure technique. This method has since been extended to a variety of other copolymer systems including polystyrene-*b*-poly(2-vinylpyridine)-*b*-poly(t-butyl methacrylate) [84], polystyrene-*b*-polyisoprene [85] and most recently polyisoprene-*b*-polylactide [86]. The most extensive work in this area has focused on the formation of a hexagonal arrangement of cylinders of the minor block component (PEO) in a PS matrix for an asymmetric PS-*b*-PEO diblock system. Russell *et al.* [87,88] have been at the forefront in refining this approach to enhance control over the lateral order in hexagonally close packed cylindrical microdomains in thin films. This technique works by lowering the glass transition temperature of the polymer because of solvent molecules that penetrate the macromolecular matrix separating the molecules, increasing the system volume and increasing mobility within the system. The lowering of the glass transition temperature as a function of even relatively small solvent content can be relatively large [89]. Although, polymer swelling apparatus can be quite complex (*e.g.*, flow-through, pressure control) it can be effected by simple placement of samples in a beaker and exposing them to a solvent vapour pressure. The advantages of the methodology can be shorter processing temperatures and enhanced thermal separation from the order-disorder temperature.

The rationalisation of solvent effects in these systems can be achieved using the concept of solvent parameters introduced earlier. The solvent parameter arose from Hildebrand and Scott’s work on describing a series of rules to explain the solubility of solids and gases and as such can be used to define whether a substance in any physical sate is soluble in a solvent [90]. In Hildebrand’s original work, the solvent parameter was used as a measure of the cohesive energy which has fundamental importance because it is a direct measure of the strength of the chemical bond holding the molecules together in the liquid. This chemical bond strength is given directly by the square of the solvent parameter which yields the cohesive energy density (as detailed above). The cohesive energy density was originally described as the internal pressure of a fluid as used in the classic non-ideal gas equation and for small molecules (that obey Raoults’s Law in similarly simple solvents) can be approximated by the heat of vaporisation per unit volume. In this way the solvent parameter is a direct measure of the intermolecular interactions that exist in a fluid and in turn explains the series of physical properties described as colligative. By addition of a solute to a solvent the intermolecular forces between solvent molecules are disrupted and thus any properties dependent on these weak chemical interactions will be altered (to a first approximation) by amounts proportional to the amount of solute not specific chemical interactions between the solute and the solvent molecules. The description of the solvent parameter given here shows that these colligative properties relate to establishment of the chemical equilibrium liquid solution and the pure solvent. Colligative properties such as vapour pressure, freezing point depression, boiling point elevation and osmotic pressure are thus defined as those properties that do not depend on the nature of the solute but only on the amount added.

The practical importance of the solubility parameter is that it allows prediction of whether one material is soluble in another. This is because unless the type and magnitude of the intermolecular interactions between different molecules in solution are similar there will be no tendency for the solute and solvent molecules to intermix and thereby reduce the free energy of the system. The tendency for solvation is often described on the basis of “complementary matching” and an interaction parameter χ defined by expressions equivalent to Equation 12. It is generally accepted that for solubility the χ value must be less than 0.5 or the differences in solubility parameter no greater than about 5 (J cm^−3^)^1/2^. When a BCP is placed in a solvent careful consideration of the solvent chemistry must be considered. Where the blocks are chemically distinct and one block has a solubility parameter close to that of the solvent, micellisation may occur although this is a very complex process [5]. Since these properties are colligative, the BCP will only truly dissolve in the solvent if the total solubility parameter (given by a simple volume ratio of the blocks and their individual δ values) is close to the solubility parameter of the solvent. However, it should be remembered that solubility parameters, since they depend on the intermolecular forces between molecules, can be strongly dependent on temperature. This coupled to low solution concentrations (such that the concentration is below the critical micelle concentrations [5]) can ensure that solutions are not micellised even where δ values might suggest this is possible.

**Figure 6.**
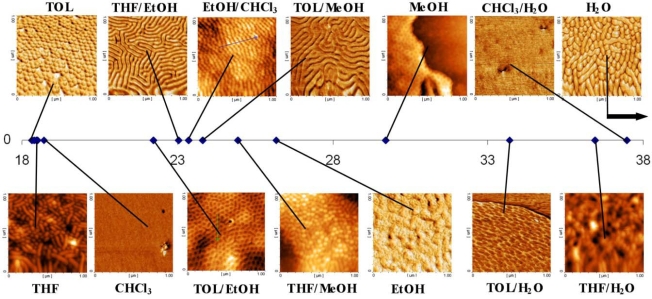
Tapping mode AFM images of 60 nm (average film thickness) thick PS-*b*-P4VP thin films following film deposition and annealing in various solvents and mixtures. The solvent parameter (MPa^1/2^) ranges from 18 to 38 (water=48).

Polystyrene-*b*-poly(4-vinylpyridine) (PS-P4VP) is one system where strong micellisation is known to occur [91]. The micelles formed are stable enough to survive solvent removal during thin film formation. Figure 6 represents a series of AFM images of a symmetric PS-*b*-P4VP block copolymer (Polymer Source Inc.) of composition Mn^PS^ = 20,000 g mol^−1^, Mn^P4VP^ = 19,000 g mol^−1^ dissolved in toluene, spin-coated onto a Si(100) surface and then solvent annealed in a series of solvents and solvent mixtures such that a range of solvent parameters could be probed. Microphase separated structures are only observed upon solvent annealing in toluene/ethanol, tetrahydrofuran/ethanol, ethanol/chloroform and toluene/methanol mixtures that closely match the solvent parameter of the BCP (~23.0 MPa^1/2^). In all other cases the patterns seen are typical of hemispherical micelles and related morphologies on the substrate. It should be noted that PS-P4VP is molecularly dispersed in this solvent [92]. These data demonstrate that choice of solvent and a fundamental understanding of the chemical interactions between the solvent and the components of the BCP are pivotal in determing the ideal conditions for generation of long range order in microphase separated BCP systems.

In a similar way that the chemical interactions of the BCP with the substrate surface can define orientation of features, the same type of control can be exerted by the surface-solvent interactions particularly in solvent annealing processing of these films. Because of the sensitivity of the BCP structure to even small changes in solvent parameter even relatively small changes in annealing conditions can cause dramatic changes in film structure. Poly(styrene-*b*-ethylene oxide) (PS-*b*-PEO) is a system which has been shown to be amenable to both orientational control and improvement in long-range ordered phase separation by the solvent annealing method [87,88]. Russell and his co-workers have explained a vertical orientation of cylinders in this system as a result of a ‘field’ effect where the evaporation of solvent molecules during solvent annealing leads to preferable vertical orientation of the cylinders. Recent work by our group demonstrates that orientational effects can also be controlled by careful control of the solvent parameter [93]. In this work it was suggested that the change in solubility parameter of a toluene/water mixture with temperature (due to changes in the vapour pressure of each of the co-solvents with temperature) was sufficient to select between an atmosphere that favoured both blocks at the surface (defining a vertical cylinder orientation) to one that favoured one block only (favouring a wetting layer of one selective block and a parallel orientation).

### 3.3. Solvent Casting Effects

**Figure 7.**
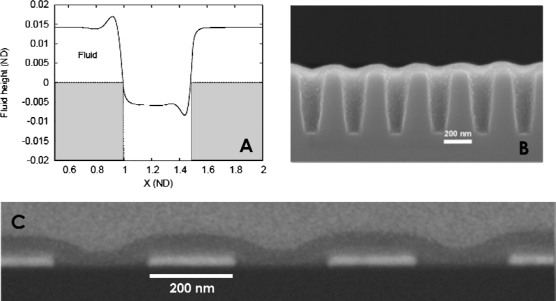
A—theoretically predicted model of polymer film development via spin-coating at an isolated topographically patterned channel in a substrate surface. The grey areas indicate the channel walls. B and C show sinusoidal variations in block copolymer polymer film thickness as observed by cross-sectional electron microscopy. B—PS-*b*-PI-*b*-PS spin-coated at deep trenches in a silicon substrate (SEM). C—PS-*b*-PEO at shallow trenches formed by creation of gold lines at a silicon substrate (TEM).

It should also be noted that the structure of films can be strongly dependent on the method of deposition, for example, in the case of spin coating onto topographically patterned surfaces. The practice and theory of spin-coating onto flat substrates is well-established [94,95] and the methodology is used practically to produce conventional photoresist thin films [96] and in more complex polymer systems [97]. As described below, regular topographically patterned substrates have considerable application for the selective alignment of polymer nanostructures in one azimuthal direction. However, the effects of this surface topography on the morphology of polymer films by spin-coating have been little explored. Recent theoretical work has suggested that substrate topography can have an important effect, giving rise to capillary waves and ridges [98]. Theoretical modelling of a typical polymer thin film formed by spin-coating solution across an isolated channel formed on a flat substrate surface is shown in Figure 7 [99]. The edge of the channel gives rise to positive film excursions in the film height at the channel wall and negative excursions in the polymer thickness within the film. Where a number of channels are placed closely together, the result is a sinusoidal variation in film thickness across the topography and this can be seen experimentally as shown in Figure 7B and 7C [99]. Because of the variation in thickness across the surface and the effect of thickness on feature orientation in microphase separated thin films, this can have a profound effect on structure [99]. Careful consideration to these effects will need to be considered because such film morphologies will make pattern transfer a difficult process.

## 4. Alignment of Microphase Separated BCP Features

**Figure 8.**
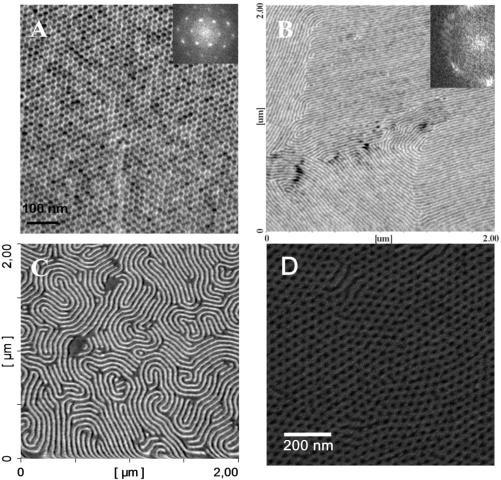
Tapping mode AFM phase images (A, B and C) and SEM (D, following selective etching to remove one block) showing typical microphase separated BCP structures formed in 50 nm thick films at silicon substrates. A shows cylindrical arrangement of PS-*b*-PEO with the PEO cylinders orientated perpendicular to the surface. B is the same material with a parallel (to the substrate surface) arrangement of cylinders. C is a polystyrene-*b*-polymethylmethacrylate (PS-*b*-PMMA) lamellar system and D is a PS-*b*-PMMA cylinder forming system with a vertical alignment of PMMA cylinders vertical to silicon substrate surface. In C and D a random PS-*b*-PMMA neutral brush layer as described earlier was used to provide a neutral wetting layer. Grain boundaries between areas of uniform alignment can be readily observed.

The microphase separation of BCPs produces regular structures but the size of the region where the pattern formed displays high regularity is dependent on the thermodynamics and kinetics that describe the phase separation process. Often the patterns have ‘domain’ like morphologies where each domain maps a region where the phase separated microstructure has a well-defined arrangement that is highly periodic. The neighbouring domains will have the same periodic microstructures but within each domain alignment of features will be random. Domain sizes can range from tens of nm to micron dimensions. Typical examples are given in Figure 8. The presence of grain boundary type structures can arise for three main reasons. Firstly, during microphase separation, the ordered structure will nucleate at many points and these ordered regions will grow until a grain boundary wall is formed. Secondly, extrinsic defects such as substrate defects or impurities can precipitate domain structures. Finally, there will be an equilibrium number of all types of defects governed by the thermodynamics (as described below). For non-equilibrium structures (arising from defects and/or growth mechanisms) the grains can be extended (coarsened) by annealing and defect annihilation. However, domain size increase will occur only slowly and has been shown to follow a t^0.25^ power law [101] and for practical purposes (*i.e.*, extended process time) it is unlikely that thermal annealing could be used to generate a single macroscopic sized domain. Further, the alignment of features would be random. For practical use in electronics the feature alignment direction must be strictly controlled to allow subsequent lithographic steps to define device and interconnect structures. Thus, in recent years, control of alignment in these nanopatterns has been of pivotal importance.

### 4.1. Pattern Alignment Control

Several methods have been adapted to control feature alignment direction in thin films of these systems. Here, alignment is defined relative to an azimuthal direction on the substrate. This alignment control is often referred to as directed self-assembly and has been reviewed several times [52,71,102,103]. The techniques used to effect alignment control of the microphase separated structures include electric fields [104–106]. With careful design of the electrodes used to generate the applied fields the films can show alignment areas of >1 cm^2^ [107]. Flow fields have also been widely used and shear forces have been shown to preferentially align structures [108–110]. Long-range alignment aided by a directional crystallisation process has also showed significant promise for this application [111]. In this technique a molten crystalline organic material is used as the solvent for the BCP and during processing the polymer microdomains align in the direction of growth of the organic crystallites [112]. However, two techniques have become dominant because the methodology may be transferable to industrial environments for generation of large area aligned nanopatterns. These are chemical patterning and graphoepitaxy.

### 4.2. Chemical Patterning

Chemical patterning, as the name suggests, involves pre-patterning the substrate surface with chemical functionality that selectively chemically interacts with one block. The microphase separated structure will then tend to align to the pre-pattern. Provided that the feature size of the chemically patterned substrate surface is matched to the feature size of the polymer pattern then the chemical interactions between the blocks and the substrate can be such as to produce almost ideal pattern of polymer. If the pattern dimensions are not matched then the surface will form defects to minimise the chemical strain. The incommensurability of the BCP feature size and the pre-pattern has been explored [114] and evolution of unusual structures shown [115]. The laboratories of Russell and Nealey pioneered this approach. Russell used a form of metal-metal oxide striped patterned substrates (formed by oblique metal deposition onto grooved surfaces) to demonstrate the possibility for preferred alignment control in PS-*b*-PMMA lamellar forming systems [116]. Nealey extended this work significantly by developing chemical patterns based around various forms of lithography to generate exceptionally well-ordered and aligned BCP microphase separated structures. Initially, extreme ultraviolet interferometric lithography was used to pattern a self-assembled monolayer of octadecyltrichlorosilane and other similar SAMs [117]. The radiation causes the formation of hydroxyl and aldehyde linkages in the SAM modifying the chemical activity in the exposed region [118]. Using x-ray interference lithography to chemically modify a PS-*r*-PMMA brush layer in a patterned manner, the Nealey group were able to show almost defect free alignment of lamellar forming PS-*b*-PMMA [119,120]. These lithographic approaches to chemical patterning show that the sort of feature size and structure requirements for generation of future generations of electronic circuitry can be attained *via* these bottom-up methodologies. However, since they are based around a pre-patterning method which in itself needs advanced lithographies, the usefulness and practicality of these combined techniques requires careful attention and already advances are being made. Cheng *et al.* used a technique known as ‘sparse’ chemical patterning where the frequency of the chemical pattern is increased by subsequent BCP patterning [120]. Ruiz *et al.* have also successfully used this type of method [121]; however an important advance reported was the use of an e-beam to write the sparse patterns [12] and this makes a significant contribution in simplifying the ease of processing these structures for potential delivery of a manufacturable technology.

### 4.3. Graphoepitaxy for Patterning

The other method used to generate highly ordered BCP nanopatterns with controlled alignment is graphoepitaxy where surface topography is used to direct the BCP structure. The term graphoepitaxy was originally coined to describe how a substrate topographic periodicity can be used to control the crystallographic alignment of thin films [122] and the technique evolved to become a popular method for defining highly crystalline polymer films [123]. It is generally accepted that strain imposed by the topography is the origin of the alignment effects, however, chemical interactions of the BCP with the topography (as outlined below) also play an important role in influenceing the alignment process. The advantage of graphoepitaxial techniques for BCP nanopattern development is that a single relatively large substrate feature such as a channel can be used to direct the BCP nanopattern with precise alignment into almost single crystal-like periodicity within the topographically defined feature. Fasolka *et al.* were the first researchers to show that corrugated substrate surfaces could be used to direct the development of microphase separated block copolymers [77]. These authors used a simple off-cut silicon substrate to generate a saw-tooth topography and this was sufficient to generate regular BCP periodicity. Segalman was the first author to demonstrate that designer topography (in this work channels or rectangular cross-section separated by flat terraces or mesas and examples provided here will refer only to this shape) could be used to generate aligned, to the edge of the channel, nanopatterns of extremely high periodicity [124]. Segalaman’s ground-breaking work not only demonstrated the possibility of this methodology for control of BCP structures but also reported the possibility of unusual edge effects due to varying film thickness as well as proposing a mechanism for alignment. It was suggested that alignment occurs *via* nucleation at the channel walls and that, below a critical channel width, a single domain structure could be formed.

Segalman’s original work was based around aligned sphere forming polystyrene-*b*-poly (2-vinylpyridine) (PS-*b*-PVP) diblock copolymers. The work has progressed very quickly and reached a high level of sophistication (see for example the review by Segalman [71]). Work reported to date has demonstrated alignment of both horizontal and vertical orientations of cylinder forming systems [52,56,126,127] and sphere forming [124,128,129] systems. One area of considerable importance has been the development ‘sparse’ surface topographies which minimise the size of the topographical features and considerably reduce the mesa contribution. Ross and co-workers have developed this technique to align vertical cylinders or spheres so that a low density of ‘posts’ guide the structure whilst being almost indistinguishable in terms of position, size and chemistry from a feature in the BCP nanopatterns [130]. These sphere and vertical cylinder structures can be used to create column or nanodot structures by pattern transfer or templating methods.

In terms of emerging electronic structures or interconnect arrangements, the formation of parallel nanowires at a substrate has become an important challenge. So-called FIN-FET structures consisting of several nanowires controlled through a single gate has become an important topic of research [131]. There has, therefore, been considerable work on the controlled alignment of lamellar (stripes orientated vertically to surface plane, Figure 3B) and cylinder (parallel to surface plane, Figure 3D) forming BCPs where coupling these techniques with templating and/or pattern transfer can transform these structures into nanowire arrays. PS-*b*-PMMA is of particular interest because of the etch characteristics of this system [132]. This has provided the motivation of much of Nealey’s work outlined above in terms of chemical patterning. However, precise control of alignment using graphoepitaxy is not as facile for these structures as for the vertical cylinder and sphere geometries.

**Figure 9.**
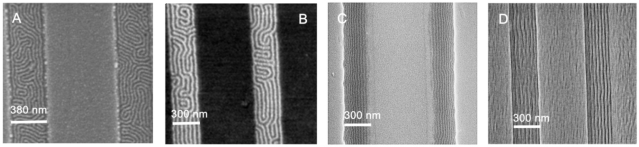
Lamellar (A) and hexagonal cylinder (B, C and D) forming arrangements of PS-*b*-PMMA in topographically patterned substrates. See text for details.

Authors have ascribed the preferential alignment of topographical patterns due to *e.g.*, the flow forces developed as material is drawn from the substrate surface into channels and grooves [133], however, it is now apparent that wetting phenomena at walls, grooves and wells dominate any such process particularly at equilibrium [132,134]. For the lamellar stripe forming systems ensuring the vertical alignment of lamellae requires surface neutralisation. However, application of the brush also neutralises the walls and no preferred alignment of the blocks parallel to the channel wall occurs as shown in Figure 9(A).

Various strategies for circumnavigating these problems have been suggested including side wall modification [134] and using cylinder forming PS-*b*-PMMA at the base of the channel to form a chemical pattern to guide the lamellar phase [135]. In a similar way, the cylinder forming systems also require interface tailoring however, this is somewhat easier because of the ideal parallel orientation and alignment is favoured by modifying both the base of a feature and the sidewalls to be selective to the majority block. This is shown explicitly in Figure 9. In Figure 9B the hexagonal cylinder forming PS-*b*-PMMA (PS as the major block) shows little alignment in channels created in silicon substrates. However, by creating the channel structure in a siloxane surface (Figure 9C) and Si_3_N_4_ (Figure 9D) PS wetting at the surface interfaces is favoured and alignment is improved.

**Figure 10.**
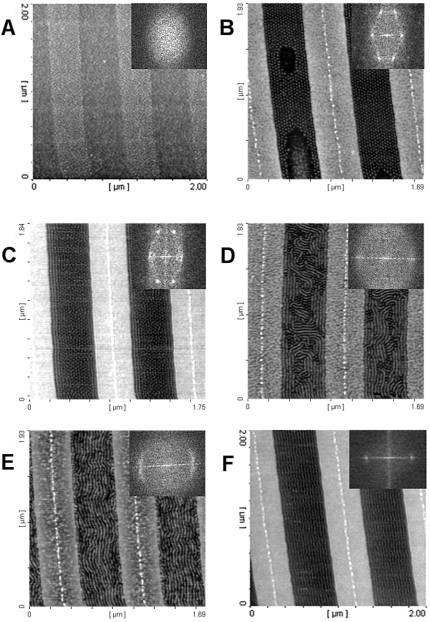
2D Tapping mode AFM phase images of PS-PI-PS thin films prepared from 0.7 wt% solutions of polymer in toluene on channel cut topographically defined substrates (433 nm channels) after annealing at about 400 K for (a) 0 h, (b) 1 h, (c) 1½ h, (d) 2 h, (e) 2½ h and (f) 3 h.

It is also pointed out that forming parallel (to the surface plane) cylinder arrangements offers some further challenges compared to vertical arrangements. Film thickness and transfer of polymer to the channels can present a number of experimental difficulties. Suh *et al.* [76] studied orientation effects in cylinder forming block copolymer films in detail and it is now generally accepted that vertical orientation of the cylinders is favoured for very thin films because the parallel arrangement can only be sustained with inclusion of elastic strain in the structure at low dimension (this strain effectively reduces with thickness). This is important because only single layers of cylinders are required if the pattern is to be used for creation of devices since multilayer structures can not be readily filled or transferred to the substrate by etch methods. Some recent data (Figure 10, AFM tapping mode phase images) show the importance of the relationship between film thickness and orientation in topographical substrates. The cylinder forming PS-*b*-PI-*b*-PS system on Si(100) substrates has a strong tendency to form parallel arrangements of cylinders because chemical interactions with both the substrate and the surface interface strongly favour PS wetting layers [136]. If sufficient BCP is spin-coated onto a channel cut surface to just fill channels (433 nm, 60 nm deep) so as to form a single layer of PI cylinders (light lines) in a PS matrix the as coated surface (Figure 10A) is an isotropic distribution of polymer across mesas and channels. Heating at about 400 K brings about phase separation and transfer of material from the mesas into the channels. After 1 h the material has formed a vertical cylinder arrangement in the channels (Figure 10B). Although the parallel arrangement is favoured by interface effects, teh strain energy associated by not being able to form a complete layer of cylinders leads to a vertical arrangement [76]. Further heating periods lead to more complete filling of channels and eventual formation of a well ordered parallel arrangement of cylinders (Figure 10F). It is important to note that alignment and orientation is nucleated at the channel edge (Figure 10C) presumably because of polymer interactions with the side wall. The alignment also proceeds via a disordered a structure as can be seen in Figure 10D and E. It is clear from these data that mass transport and redistribution of polymer (towards an equilibrium distribution) requires careful study on these topographic surfaces.

## 5. Defect Formation

In a self-assembled structure there are likely to be reasonable concentrations of defects. This is suggested in Equation 1, ΔG_SA_ = ΔH_SA_ – TΔS_SA_, because in most cases the thermodynamic driving force for self-assembly is provided by weak intermolecular interactions and is usually of the same order of magnitude as the entropy term. Practically, for any self-assembling system to reach the minimum free energy configuration there must be enough thermal energy to allow the mass transport of the self-assembling moieties [1]. In these circumstances, obtaining defect free self-assembly over macroscopic areas is improbable. A self-assembled nanopatterned surface is likely to show a number of distinct irregularities or defects and these can take many forms as outlined below. The origins of these defects are manifold but each defect comes with an energy cost because it disrupts the arrangement of the polymer blocks which provides a free energy minimum within the film.

### 5.1. Intrinsic Defects in Block Copolymer Nanopatterned Surfaces

An intrinsic defect in a thin film can be defined a defect that is formed within the film without undue influence of the substrate. The formation of a perfect pattern is reliant on favourable thermodynamic limitations and kinetics. At any temperature there will be an ‘equilibrium’ concentration of defects governed by a Bolzmann distribution. There will also be a ‘non-equilibrium’ (or kinetic) concentration of defects as pattern formation is reliant on material mass transport and this may require process times beyond experimental limitations and defects may be ‘frozen’ in.

#### 5.1.1. Equilibrium Defects

As above, the thermodynamically defined concentration of defects originates from a balance of configurational entropy and the energy cost associated with the defect. These defects are ‘statistical’ in nature and while individual defects may have limited lifetimes a population of them will always exist at a concentration defined by conditions.

The thermodynamics of defects in fully equilibrated systems is well understood but care must be taken to separate the free energy defining self-assembly and pattern formation from the free energy of defect formation so that the role of intermolecular forces can be well understood. For defect formation the free energy of single defect formation is given by

(19) $${\Delta \text{G}}_{\text{DF}}={\Delta \text{H}}_{\text{DF}}-{\text{T}\Delta \text{S}}_{\text{DF}}$$

The enthalpy term, ΔH_DF_, does not necessarily reflect the intermolecular forces between blocks—it is the energy cost associated with disrupting the pattern and may be thought of as a region where optimum arrangement does not occur and the reduction of enthalpy associated with ideal self-assembly is not realised. For example, a system of hexagonally packed cylinders may exhibit defect regions of lamellar structure. The enthalpy of defect formation is partially related to the enthalpic difference between the two structural arrangements and this might be much less than the intermolecular forces between blocks. If the difference in enthalpy of two different arrangements is small a relatively high equilibrium concentration of defects might be expected compared to one where the enthalpy difference is large. The entropy difference now reflects the order change between the prefect and defective structural arrangement. Note here that the enthalpy cost of creating a defect is not only determined by the respective differences in structural arrangement but also by strain energy (due to tensile and compressive forces that are associated with defect insertion) as well as any interfacial effects arising from intermolecular interactions in the areas around the defect. If ΔG_DF_ is negative there will be a finite number of defects in the system and the concentration will be given by

(20) $$\text{N}/{\text{N}}_{0}=\text{exp}\;(-{\Delta \text{E}}_{\text{act}}/\text{RT})\;[137]$$

N is the number of defects in a matrix of N_0_ self-assembled moieties or features and ΔE_act_ is the activation energy of defect formation. The activation energy ΔE_act_ should not be confused with ΔH_DF_. The activation energy represents energy difference between the initial ideally arranged state and a transition state towards the defective structure.

Equation 19 can be used to estimate the defect concentration by use of the Boltzmann formula to estimate entropy S [137]

(21) S=klnW

where W is the number of ways of arranging n defects over N possible features within a system of periodic structure. W can be written

(22) W=N!/((N-n)!n!)

and using Stirling’s approximation we can write that

**Figure 11.**
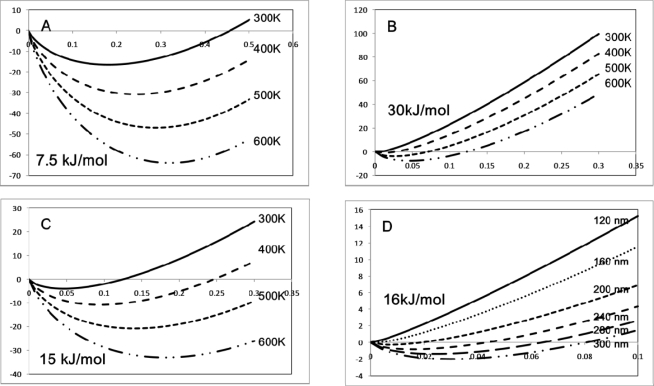
(A)–(C) Free energy of defect formation as a function of defect concentration (as ratio of defects to total number of features) for three different activation energies for defect formation. (D) Free energy of defect formation versus defect concentration at various channel widths.

(23) ln N!∼N ln N−N

so that

(24) $${\text{T}\Delta \text{S}}_{\text{DF}}=\text{kT}[\text{N}\;\text{ln}\;\text{N}-\text{n}\;\text{ln}\;\text{n}-(\text{N}-\text{n})\;\text{ln}\;(\text{N}-\text{n})]$$

and

(25) $${\Delta \text{G}}_{\text{DF}(\text{n})}={\text{n}\Delta \text{H}}_{\text{DF}}-\text{kT}[\text{N}\;\text{ln}\;\text{N}-\text{n}\;\text{ln}\;\text{n}-(\text{N}-\text{n})\;\text{ln}\;(\text{N-n})]$$

where ΔG_DF(n)_ represents the free energy cost of forming n defects in the system. This formulism allows a plot of free energy against n/N, the defect ratio, as shown in Figure 11. Three plots (Figures 11A–C) are shown for defect formation energies of what might be expected for a BCP system *e.g.*, Hammond *et al.* [138] have measured the defect formation energy in a PS-*b*-P2VP system at around 30 kJ mol^−1^. Although these are simple calculations they illustrate the salient features of equilibrium defect formation. At low defect concentrations defect formation is entropy driven until a critical concentration of defects allows the activation energy term to compensate for entropy. There is usually an equilibrium defect density indicated at the minimum free energy. As might be expected, as the activation energy for defect formation increases this equilibrium defect density. At high activation energy values (*e.g.*, 30 kJ mol^−1^) and low temperature (300 K) there is no thermodynamic driving force for defect formation and the data suggests that in BCP systems it should be possible to form highly regular structures.

#### 5.1.2. Non-Equilibrium Defects

Practically, there are few examples of defect free microphase separation of BCP thin film systems. As these films are normally prepared by non-equilibrium methods such as spin- or dip-coating the microphase separated structure evolves by either thermal or solvent annealing and defects are introduced (nucleation of microphase separated regions) or removed by growth kinetics. Through the annealing cycle phase separated regions will nucleate in various places, grow and increase in order [138–141].

Due to the nature of the chemical interactions between blocks and the relatively high glass transition temperatures coupled to low meting points and low order-disorder temperatures, the temperature window for annealing out these non-equilibrium defects may be rather small and the defects may be essentially kinetically stable). Extended heating times well-separated from the order-disorder temperature may be required to remove defects and practically (because of local and large area mass transport limitations) equilibrium may not be achieved even after inordinately long annealing periods and non-equilibrium defects will be present. This is largely due to the requirement for defect annihilation associated with coarsening of the randomly orientated grain structure that results from the kinetics of nucleation and growth of phase separated regions [101]. Thus, although ΔG_DF_ may be positive for many BCP systems, implying no defects should be formed if the system attains complete equilibrium, in practice this is unlikely. In many cases a clear distinction of equilibrium defects and non-equilibrium defects can not be practically achieved. The advent of advanced force microscopy methods facilitates defect studies without causing damage to the sample [142–144]. Of particular importance are *in situ* AFM methods that allow real time data collection during pattern evolution [144].

### 5.2. Extrinsic Defects in Block Copolymer Nanopatterned Surfaces

It is highly unlikely that a true minimum energy configuration of a BCP film on a flat substrate (which may be a defect free arrangement) can be achieved in practice because of the effects outlined above. However, if the pattern can be aligned to a substrate feature (through favourable chemical interactions) then random grain orientations can be avoided and the requirement for defect annihilation during grain coarsening can be removed and ideally ordered arrangements may be achieved. Graphoepitaxy and chemical patterning as described above offer a means of aligning patterns and thus potentially provide a solution to problems associated with forming defect free films. It thus seems necessary that these bottom-up techniques for patterned surface formation are combined with a top-down lithographic method in order to achieve ideal arrangements. Chemical pre-patterning of surfaces has shown great potential in this regard [119–121,145]. The main drawback of the chemical patterning methodology is a requirement to pre-pattern on the same length scale as the phase separated BCP feature size and this requires very advanced lithographic methods. One innovative approach to the problem of developing and use of advanced lithography is to use the assembly of another block copolymer film which can be readily aligned through favourable interactions with substrate features (see below). This polymer film is then subsequently used to chemically pattern the BCP of interest. This approach has been used with a cylinder forming PS-*b*-PMMA system to form a chemical pattern for development of well-ordered lamellar forming PS-*b*-PMMA [135].

Low defect concentrations in BCP phase separated structures have been reported using graphoepitaxial methods. As discussed above, in favourable circumstances the topographically patterned surfaces align and orientate the phase separated BCP structure through interactions between the surfaces and one or both blocks. These interactions force the BCP structure into registry and single grain structures. There are many examples in the literature of graphoepitaxial defined single grain structures [126–128,132,135,136,146–151]. Various authors report that the defect nature of these directed structures are largely insensitive to the match between polymer feature spacing and channel width (commensurability) except that as width increases there is a corresponding increase in the number of polymer features within the topography. It is of course noted that the polymer structure will exhibit strain (*i.e.*, the spacing between features will stretch or compress) so as to fit an integer number of features across the topography such that strain is minimised. As shown by Ross and co-workers [128] the energy of the system will be minimum at the trench width at which commensurability occurs (*i.e.*, width = n polymer feature spacings, n = an integer) but will increase at higher or lower values. The strain energy will be at a maximum when the trench width is equivalent to n + ½ polymer feature spacings).

**Figure 12.**
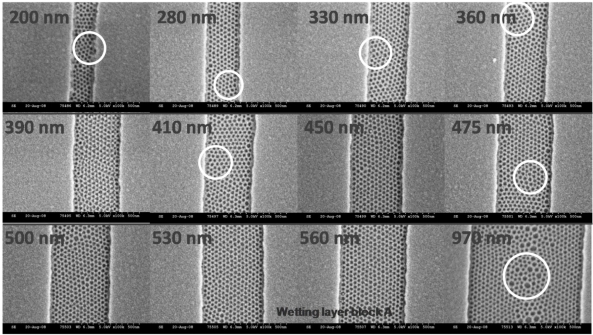
SEM images of etched cylinder forming PS-*b*-PEO (PEO removed) in rectangular trenches (60 nm depth and width as shown) of various widths. The white circles show various defects present including grain boundaries, dislocations and point defects. See text for further details.

However, Nealey has noted that the defect concentration within the phase separated polymer structure is highly dependent on the degree of commensurability with a strong correlation observed for the cylinder forming PS-*b*-PMMA system. We observed similar effects in the cylinder forming PS-*b*-PEO system as shown in Figure 12 and defect free single grain structures were a rarity except at obviously commensurate trench widths (*e.g.*, 390 and 560 nm). The difference between these studies and other systems probably lies in the deformability of the polymer blocks. Analysis of the data in figure 12 suggests a number of other points. Firstly, relatively small amounts of strain around narrower trench widths can relatively easily precipitate defects and defect concentrations are relatively high. As the width increases the defect concentration observed apparently decreases until at very high widths it increases again. We explain these observations in the following manner. The BCP energy is dominated by block-block and block-interface interactions so that filling of the topography and maximising the number of features within the topography are the most important factors. When the channel width and phase separated feature spacing is incommensurate, strain energy (highly dependent on the polymer properties) results and an ideal pattern can only be achieved if this strain energy is less than the total energy recoverable from changes in structure and defect formation. Thus, as width increases it becomes easier to maintain ideal single grain, defect free structures because the strain is distributed over a larger polymer volume and so is proportionally less. At very large channel widths nucleation can occur at both side walls leaving an area at centre where defects must form to allow volume fill.

**Figure 13.**
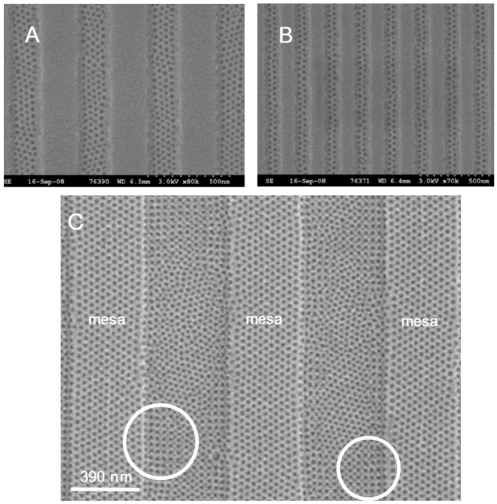
SEM images of etched cylinder forming PS-*b*-PEO (PEO removed) in rectangular trenches (60 nm depth and widths of 175 (A), 120 (B) and 390 (C) nm). Polymer was deposited to just fill channels in (A) and (B) but was overfileld in (C) to allow phase separation at the mesas.

If this description is correct there should be a minimum channel width where no equilibrium defects should be observed since the introduction of strain energy would raise the total energy of the system excessively. This can be modelled in the same way as described above except that the activation energy for defect formation (a value of 16 kJ mol^−1^ was used) is effectively increased by an incommensurate strain energy of 4 kJ mol^−1^. It was further assumed that this incommensurate strain was dispersed over the volume of polymer in the channel. The results are shown in Figure 11(D) which illustrates that even when defect formation is favourable, that there is a critical dimension when there should be no equilibrium defects formed. Figure 13 provides experimental support for this model. In the narrowest channel widths of 20 nm, there is an almost ideal arrangement of BCP structure. Figure 13(C) provides clear evidence that the majority of defects observed in graphoepitaxy result form the strain introduced by the channels. Here, a cylinder forming PS-*b*-PEO polymer was spin-coated to create material at the channel mesas and within the channel. The BCP structure within the channel is highly defective with the defect motifs seen above clearly visible. Also the expected hexagonal pattern is not exhibited uniformly and an unexpected cubic arrangement is observed in places as a result of the imposed strain. However, at the mesas, an almost ideal structure is seen. This can be explained by the fact that any strain caused by incommensurity can be relived by a slight expansion of the film.

## 6. Concluding Remarks

The microphase separation of block copolymers shows a great deal of promise as a means of generating regular nanopatterns at surfaces. They may, therefore, find application as a means to novel nanomaterials and nanoelectronics device structures. The possible formation of these patterns is thermodynamically determined by the strength of the chemical interactions which is balanced by entropy considerations. Polymer composition determines the structural arrangement whilst molecular weight and the physical properties determine the kinetics of the phase separation process. However, in thin film form the chemical interactions between the blocks and their environment, *i.e.*, the interfaces that surround them, must also be carefully considered so that the microphase structure exhibits controlled alignment and orientation. The use of surface engineering to control the chemical interactions with the surface and chemical pre-patterning are strict needs if the requirements for long-range order and periodicity are to be met. Like all self-assembly processes, defect chemistry is important and these systems can exhibit a number of defects originating from thermodynamic and kinetic limitations. However, recent work suggests that defect-free patterns over macroscopic dimensions may be achievable.

## References

[1] Whitesides GM Grzybowski B Self-assembly at all scales Science 2002 295 2418 2421 11923529 10.1126/science.1070821

[2] Whitesides GM Mathias JP Seto CT Molecular self-assembly and nanochemistry: A chemical strategy for the synthesis of nanostructures Science 1991 254 1312 1319 1962191 10.1126/science.1962191

[3] Murray CB Kagan CR Bawendi MG Self-organization of CdSe nanocrystallites into three-dimensional quantum dot superlattices Science 1995 270 1335

[4] Schwarz DK Mechanisms and kinetics of self-assembled monolayer formation Annu. Rev. Phys. Chem 2001 52 107 137 11326061 10.1146/annurev.physchem.52.1.107

[5] Svenson S Controlling surfactant self-assembly Curr. Opin. Colloid Interface Sci 2004 9 201 212

[6] Kresge CT Leonowicz ME Roth WJ Vartuli JC Beck JS Ordered mesoporous molecular sieves synthesized by a liquid-crystal mechanism Nature 1992 359 710 712

[7] Holmes JD Morris MA Ryan KM Self Assembly—The Future1st ed Robinson BH Elsevier Amsterdam, The Netherlands 2003 Chapter 2 175

[8] Misteli T The concept of self-organization in cellular architecture J. Cell Biol 2001 155 181 185 11604416 10.1083/jcb.200108110 PMC2198832

[9] Care CM Dalby T Packing entropy in micelle self-assembly Europhys. Lett 1999 45 38 42

[10] Yodh AG Lin K-H Crockery JC Dinsmorez AD Verma R Kaplan PD Entropically driven self-assembly and interaction in suspension Phil. Trans. R. Soc. Lond A 2001 359 921 937

[11] Huie JC Guided molecular self-assembly: A review of recent efforts Smart Mater. Struct 2003 12 264 271

[12] Neidle S Oxford Handbook of Nucleic Acid Structure Oxford University Press Oxford, UK 1999

[13] Ninham B Self Assembly Robinson BH IOS Press Amsterdam, The Netherlands 2003 11

[14] Würthner F Perylene bisimide dyes as versatile building blocks for functional supramolecular architectures Chem. Commun 2004 14 1574 10.1039/b401630k 15263926

[15] Olenyuk B Whiteford JA Stang PJ Self-assembly of nanoscale cuboctahedra by coordination chemistry Nature 1990 398 796 799 10235260 10.1038/19740

[16] Lindgård P-A Reflections on the protein-folding problem J. Phys.: Condens. Matter 2003 15 S1779 S1786

[17] Israelachivili J Intermolecular and surface forces2nd ed Academic Press NY, USA 1991 1 130

[18] Leclère Ph Surin M Brocorens P Cavallini M Biscarini F Lazzaroni R Supramolecular assembly of conjugated polymers: From molecular engineering to solid-state properties Mater. Sci. Eng. R 2006 55 1 56

[19] D’Errico G Encyclopaedia of Surface and Colloid Science Somasundaran P CRC Press Boca Raton, FL, USA 2006 3840 3848

[20] Ryan KM Coleman NRB Lyons DM Morris MA Steytler DC Heenan RK Holmes JD Control of pore morphology in mesoporous silica synthesized from triblock copolymer templates Langmuir 2002 18 4996 5001

[21] Carraher CE Jr Introduction to Polymer Chemistry CRC Press New York, NY, USA 2007

[22] Hamley IW Developments in Block Copolymer Science and Technology Wiley Hoboken, NJ, USA 2004

[23] Lazzari M Liu G Lecommandoux S Block Copolymers in Nanoscience Wiley-VCH. Weinheim Berlin, DE, Germany 2006

[24] Kim G Libera M Microstructural development in solvent-cast polystyrene-polybutadiene-polystyrene (SBS) triblock copolymer thin films Macromolecules 1998 31 2569 2577

[25] Burke GM Woscholski R Yaliraki SN Differential hydrophobicity drives self-assembly in Huntington’s disease Proc. Nat. Acad. Sci. USA 2003 100 13928 13933 14617779 10.1073/pnas.1936025100 PMC283523

[26] Hildebrand JH The Solubility of Non-Electrolytes Reinhold Publishing Corporation New York, NY, USA 1936

[27] Burke J Solubility parameters: Theory and application AIC Book and Paper Group Annual 1984 3 13 58

[28] Barton AFM Handbook of Polymer-liquid Interaction Parameters and Solubility Parameters CRC Press Boca Raton, FL, USA 1990

[29] Hansen CM The three dimensional solubility parameter key to paint component affinities: 1. Solvents, plasticizers, polymers, and resins J. Paint Technol 1967 39 104 117

[30] Hansen CM The universality of the solubility parameter concept Ind. Eng. Chem. Prod. Res. Dev 1969 8 2 11

[31] Bates FS Polymer-polymer phase behaviour Science 1991 251 898 905 17847383 10.1126/science.251.4996.898

[32] Flory PJ Thermodynamics of high polymer solutions J. Chem. Phys 1942 10 51 61

[33] Huggins ML Theories of solutions of high polymers J. Am. Chem. Soc 1942 74 1712 1719

[34] Matsen MW Bates FS Unifying weak- and strong-segregation block copolymer theories Macromolecules 1996 28 1091 1098

[35] Leibler L Theory of microphase separation in block copolymers Macromolecules 1980 13 1602 1617

[36] Grason GM The packing of soft materials: Molecular asymmetry, geometric frustration and optimal lattices in block copolymer melts Phys. Rep 2006 433 1 64

[37] Khandpur AK Förster S Bates FS Hamley IW Ryan AJ Bras W Almdal K Mortensen K A small-angle neutron scattering study of orientational order in the nematic phase of a thermotropic liquid crystal Macromolecules 1995 28 8796 8806

[38] Melenkevitz J Muthukumar M Density functional theory of lamellar ordering in diblock copolymers Macromolecules 1991 24 4199 4205

[39] Bates FS Fredrickson GH Block copolymers—Designer soft materials Phys. Today 1998 52 32 38

[40] Grason GM Continuous crystallization in hexagonally ordered materials Phys. Rev. Letts 2008 101 105702 105706 18851228 10.1103/PhysRevLett.101.105702

[41] Hajduk DA Takenouchi H Hillmyer MA Bates FS Vigild ME Almdal K Stability of the perforated layer (PL) phase in diblock copolymer melts Macromolecules 1997 30 3788 3795

[42] Hamley IW Koppi KA Rosedale JH Bates FS Almdal K Mortensen K Hexagonal mesophases between lamellae and cylinders in a diblock copolymer melt Macromolecules 1993 26 5959 5970

[43] Knoll A Horvat A Lyakhova KS Krausch G Sevink GJA Zvelindovsky AV Magerle R Phase behavior in thin films of cylinder-forming block copolymers Phys. Rev. Letts 2002 89 035501 035505 12144400 10.1103/PhysRevLett.89.035501

[44] Castelletto V Hamley IW Morphologies of block copolymer melts Current Opinion in Sol. State Mater. Sci 2004 8 426 438

[45] Rice R Arnold DC Shaw MT Lacopina I Quinn AJ Amenitsch H Holmes JD Morris MA Ordered mesoporous silicate structures as potential templates for nanowire growth Adv. Funct. Mater 2007 17 133 141

[46] Petkov N Platschek B Morris MA Holmes JD Bein T Oriented growth of metal and semiconductor nanostructures within aligned mesoporous channels Chem. Mater 2007 19 1376 1381

[47] Pileni MP Nanocrystal self-assemblies: Fabrication and collective properties J. Phys. Chem. B 2001 105 3358 3371

[48] Lodge TP Block copolymers: Past successes and future challenges Macromol. Chem. Phys 2003 204 265 273

[49] Fasolka M Mayes AM Block copolymer thin films—Physics and applications Ann. Rev. Mater. Res 2001 31 323 355

[50] Soo PP Huang BY Jang YI Chiang YM Sadoway DR Mayes AM Rubbery block copolymer electrolytes J. Electrochem. Soc 1999 146 32 37

[51] Ulbricht M Advanced functional polymer membranes Polymer 2006 47 2217 2262

[52] Park C Yoon J Thomas EL Enabling nanotechnology with self assembled block copolymer patterns Polymer 2003 44 6725 6760

[53] Chen VZH Hoffman J Lee VY Latrou H Avgeropoulus A Hadjichristidis N Miller RD Thomas EL Ordered bicontinuous nanoporous and nanorelief ceramic films from self assembling polymer precursors Science 1999 286 1716 1719 10576734 10.1126/science.286.5445.1716

[54] Farrell RA Petkov N Cherkaoui K Hurley P Amenitsch H Holmes JD Morris MA Thin and continuous films with controlled bi- and tri-modal porosities by embedment of zeolite nanoparticles in a mesoporous Matrix J. Mat. Chem 2008 18 2213 2220

[55] Farrell RA Cherkaoui K Petkov N Amenitsch H Holmes JD Hurley PK Morris MA Physical and electrical properties of low dielectric constant self-assembled mesoporous silica thin films Microelectron. Reliab 2007 47 759 763

[56] Cheng JY Ross CA Chan VZH Thomas EL Robb GH Lammertink RGH Vancso GJ Formation of a cobalt magnetic dot array via block copolymer lithography Adv. Mater 2001 13 1174 1178

[57] Edrington AC Urbas AM DeRege P Chen CX Swager TM Hadjichristidis N Xenidou M Fetters LJ Joannopoulos JD Fink Y Thomas EL Polymer-based photonic crystals Adv. Mater 2001 13 421 425

[58] Pease RF Chou SY Lithography and other patterning techniques for future electronics Pro. IEEE 2008 96 248 270

[59] Del Campo A Artz E Fabrication approaches for generating complex micro- and nanopatterns on polymeric surfaces Chem. Rev 2008 108 911 945 18298098 10.1021/cr050018y

[60] Bloomstein TM Marchant MF Deneault S Hardy DE Rothschild M 22-NM immersion interference lithography Opt. Express 2006 14 6434 6443 19516821 10.1364/oe.14.006434

[61] Davari B Dennard RH Shahidi GG CMOS scaling for high performance and low power-the next ten years Proc. IEEE 1995 83 595 606

[62] ITRS roadmap 2005 [Online]Available: http://www.itrs.net/ (accessed August 7, 2009).

[63] Whitesides GM Mathias JP Seto CT Molecular self-assembly and nanochemistry—A chemical strategy for the synthesis of nanostructures Science 1991 254 1312 1319 1962191 10.1126/science.1962191

[64] Hillenius S Herr D Weitzman B Welser J SRC collaborations paving the way IEEE Nanotechnol. Mag 2007 1 6 12

[65] Jeong SJ Xia G Kim BH Shin DO Kwon SH Kang SW Kim OS Universal block copolymer lithography for metals, semiconductors, ceramics, and polymers Adv. Mater 2008 20 1898 1904

[66] Yamaguchi T Yamaguchi H Block copolymer lithography toward 16-nm-technology nodes and beyond NTT Tech. Rev 2006 4 17 24

[67] Stoykovich MP Nealey PF Block copolymers and conventional lithography Mater. Today 2006 9 20 29

[68] Cheng JY Sanders DP Kim HC Sundberg LK Integration of polymer self-assembly for lithographic application Proc SPIE 2008 6921 692127/1 692127/8

[69] Aizawa M Buriak JM Block copolymer templated chemistry for the formation of metallic nanoparticle arrays on semiconductor surfaces Chem. Mater 2007 19 5090 5101

[70] Krishnamoorthy S Hinderling C Heinzelmann H Nanoscale patterning with block copolymers Mater. Today 2002 9 40 47

[71] Segalman RA Patterning with block copolymer thin films Mater. Sci. Eng. R 2005 48 191 226

[72] Qi S Wang ZG On the nature of the perforated layer phase in undiluted diblock copolymers Macromolecules 1997 30 4491 4497

[73] Knoll A Lyakhova KS Horvat A Krausch G Sevink GJA Zvelindovsky AV Magerle R Direct imaging and mesoscale modelling of phase transitions in a nanostructured fluid Nat. Mater 2004 3 886 891 15568030 10.1038/nmat1258

[74] Seo YA Kim E Kwon SY Jing H Shin K AFM study of phase-separated morphology in immiscible blend thin films Ultramicroscopy 2008 10 1186 1190 18565669 10.1016/j.ultramic.2008.04.043

[75] Green PF Limary R Block copolymer thin films: Pattern formation and phase behaviour Adv. Colloid Interface Sci 2001 94 53 81

[76] Suh JY Kim YS Lee HH Parallel and vertical morphologies in block copolymers of cylindrical domains J. Chem. Phys 1998 108 1253 1256

[77] Fasolka MJ Harris DJ Mayes AM Yoon M Mochrie SGJ Observed substrate topography-mediated lateral patterning of diblock copolymer films Phys. Rev. Lett 1997 79 3018 3021

[78] Sohn BH Yun SH Perpendicular lamellae induced at the interface of neutral self-assembled monolayers in thin diblock copolymer films Polymer 2002 43 2507 2512

[79] Mansky P Liu Y Huang E Russell TP Hawker CJ Controlling polymer-surface interactions with random copolymer brushes Science 1997 275 1458 1460

[80] In I La YH Park SM Nealey PF Gopalan P Side-chain-grafted random copolymer brushes as neutral surfaces for controlling the orientation of block copolymer microdomains in thin films Langmuir 2006 22 7855 7860 16922574 10.1021/la060748g

[81] Sperling LH Introduction to Physical Polymer Science4th ed Wiley Hoboken, NJ, USA 2006

[82] Schott H Swelling kinetics of polymers J. Macromol. Sci. Part B 1992 3 1 9

[83] Fukunaga K Elbs H Magerle R Krausch G Large-scale alignment of ABC block copolymer microdomains via solvent vapor treatment Macromolecules 2000 33 947 953

[84] Niu SJ Saraf RF Stability of order in solvent-annealed block copolymer thin films Macromolecules 2003 36 2428 2440

[85] Cavicchi KA Russell TP Solvent annealed thin films of asymmetric polyisoprene-polylactide diblock copolymers Macromolecules 2001 40 1181 1186

[86] Lin Z Kim DH Wu X Boosahda L Stone D LaRose L Russell TP A rapid route to arrays of nanostructures in thin films Adv. Mater 2002 14 1373 1376

[87] Kim SH Misner MJ Xu T Kimura M Russell TP Highly oriented and ordered arrays from block copolymers via solvent evaporation Adv. Mater 2004 16 226 231

[88] Gutierrez MH Ford WT The glass-to-gel transition in solvent-swollen polystyrene networks J. Polym. Sci., Part B: Polym. Chem 2003 24 655 663

[89] Hildebrand JH Scott RL The Solubility of Nonelectrolytes3rd ed Reinhold Publishing Corporation New York, NY, USA 1950

[90] Tassin JF Siemans RL Tang WT Hadziioannou G Swalen JD Smith BA Kinetics of adsorption of block copolymers revealed by surface plasmons J. Phys. Chem 1989 93 2106 2111

[91] Cheng F Yang X Peng H Chen D Jing M Well-controlled formation of polymeric micelles with a nanosized aqueous core and their applications as nanoreactors Macromolecules 2007 40 8007 8014

[92] Fitzgerald TG Farrell RA O’Driscoll S O’Mahony CT Holmes JD Morris MA Orientation and translational control of PS-*b*-PEO/PS thin films via solvent annealing and graphoepitaxy techniques e-J. Surf. Sci. Nanotech 2009 7 741 745

[93] Scriven LE Physics and applications of dip coating and spin coating Mater. Res. Soc. Symp. Proc 1998 121 717 729

[94] Liquid Film Coating: Scientific Principles and Their Technological Implications1st ed Kistler SF Schweizer PM Chapman and Hall London, UK 1997

[95] Weill A Francou JM Dechenaux E Photoresist spin coating mechanism related to polymer solution rheology Microelectron. Eng 1987 6 427 431

[96] Yimsiria P Mackley MR Spin and dip coating of light-emitting polymer solutions: Matching experiment with modelling Chem. Eng. Sci 2006 61 3496 3505

[97] Matar OK Sisoev GM Lawrence CJ Thin film flow over spinning discs: The effect of surface topography and flow rate modulation Chem. Eng. Sci 2008 63 2225 2232

[98] Fitzgerald TG Farrell RA O’Driscoll S O’Mahony CT Holmes JD Morris MA Orientation and translational control of PS-*b*-PEO/PS thin films *via* solvent annealing and graphoepitaxy techniques e-J. Surf. Sci. Nanotech 2009 7 471 475

[99] Fitzgerald TG Farrell RA Petkov N Shaw MT Charpin J Gleeson JP Holmes JD Morris MA A study on the combined effects of solvent evaporation and polymer flow upon block copolymer self-assembly and alignment on topographic patterns Langmuir10.1021/la9018162 19860380

[100] Perlich J Körstgens V Metwalli E Schulz L Georgii R Müller-Buschbaum P Solvent content in thin spin-coated polystyrene homopolymer films Macromolecules 2009 42 337 344

[101] Harrison C Adamson H Cheng Z Sebastian JM Sethuraman S Huse DA Register A Chaikin PM Mechanisms of ordering in striped patterns Science 2000 290 1558 1560 11090350 10.1126/science.290.5496.1558

[102] Darling SB Directing the self-assembly of block copolymers Prog. Polym. Sci 2007 32 1152 1204

[103] Kim HC Hinsberg WD Surface patterns from block copolymer self-assembly J. Vac. Sci. Technol. A 2008 26 1369 1382

[104] Thurn-Albrecht T DeRouchey J Russell TP Kolb R Pathways toward electric field induced alignment of block copolymers Macromolecules 2002 35 8106 8110

[105] Amundsen K Helfand E Quan XN Hudson SD Smith SD Alignment of lamellar block copolymer microstructure in an electric field. 2. Mechanisms of alignment Macromolecules 1994 27 6559 6570

[106] Morkved TL Lu M Urbas AM Ehrichs EE Jaeger HM Mansky P Russell TP Local control of microdomain orientation in diblock copolymer thin films with electric fields Science 1996 273 931 933 8688070 10.1126/science.273.5277.931

[107] Mansky P DeRouchey J Mays J Pitsikalis M Morkved T Russell TP Large-area domain alignment in block copolymer thin films using electric fields Macromolecules 1998 31 4399 4401

[108] Angelescu DE Waller JH Adamson DA Deshpandle P Chou SY Register RA Chaikin PM Macroscopic orientation of block copolymer cylinders in single-layer films by shearing Adv. Mater 2004 16 1736 1740

[109] Kimura M Misner MJ Xu T Kim SH Russell TP Long-range ordering of diblock copolymers induced by droplet pinning Langmuir 2003 19 9910 9913

[110] Petermann J Gohil RM New method for the preparation of high modulus thermoplastic films J. Mater. Sci 1979 14 2260 2264

[111] Park C Rosa CD Thomas EL Large area orientation of block copolymer microdomains in thin films via directional crystallization of a solvent Macromolecules 2001 34 2602 2606

[112] Park C Rosa CD Lotz B Fetters LJ Thomas EL Molecular and microdomain orientation in semicrystalline block copolymer thin films by directional crystallization of the solvent and epitaxy Macromol. Chem. Phys 2003 204 1514 1523

[113] Kim SO Solak HH Stoykovich MP Ferrier NJ de Pablo JJ Nealey PF Epitaxial self-assembly of block copolymers on lithographically defined nanopatterned substrates Nature 2003 424 411 414 12879065 10.1038/nature01775

[114] Chen H Chakrabarti J Morphology of thin block copolymer films on chemically patterned substrates J. Chem. Phys 1998 108 6897 6905

[115] Pereira GG Williams DRM Diblock copolymer thin film melts on striped, heterogeneous surfaces: Parallel, perpendicular and mixed lamellar morphologies Macromolecules 1999 32 758 764

[116] Rockford I Liu Y Mansky P Russell TP Yoon M Mochrie SGJ Polymers on nanoperiodic, heterogeneous surfaces Phys. Rev. Lett 1999 82 2602 2605

[117] Peters RD Yang XM Wang Q de Pablo JJ Nealey PF Combining advanced lithographic techniques and self-assembly of thin films of diblock copolymers to produce templates for nanofabrication J. Vac. Sci. Tech B 2000 18 3530 3534

[118] Kim TK Yang XM Peters RD Sohn BH Nealey PF Chemical modification of self-assembled monolayers by exposure to soft X-rays in air J. Phys. Chem. B 2000 104 7403 7410

[119] Stoykovich MP Müller M Kim SO Solak HH Edward EW de Pablo JJ Nealey PF Directed assembly of block copolymer blends into nonregular device-oriented structures Science 2005 308 1442 1446 15933196 10.1126/science.1111041

[120] Cheng JY Rettner CT Sanders DP Kim HC Hinsberg WD Dense self-assembly on sparse chemical patterns: Rectifying and multiplying lithographic patterns using block copolymers Adv. Mater 2008 20 3155 3158

[121] Ruiz R Kang H Detcheverry FA Dobisz F Kercher D Albrecht TR de Pablo JJ Nealey PF Density multiplication and improved lithography by directed block copolymer assembly Science 2008 321 936 939 18703735 10.1126/science.1157626

[122] Smith HI Flanders DC Orientated crystal-growth on amorphous substrates using artificial surface-relief gratings Appl. Phys. Letts 1978 32 349 350

[123] Whittmann JC Smith P Highly orientated thin-films of poly(tetrafluoroethylene) as a substrate for orientated growth of materials Nature 1991 353 414 417

[124] Segalman RA Yokoyama H Kramer EJ Graphoepitaxy of spherical domain block copolymer films Adv. Mater 2001 13 1152 1155

[125] Cheng JY Ross CA Thomas EL Smith HI Vancso GJ Fabrication of nanostructures with long-range order using block copolymer lithography Appl. Phys. Lett 2002 81 3657 3659

[126] Xiao S Yang XM Edwards EW La WH Nealey PF Graphoepitaxy of cylinder-forming block copolymers for use as templates to pattern magnetic metal dot arrays Nanotechnology 2005 16 S324 S329 10.1088/0957-4484/16/7/003 21727448

[127] Chen F Akasaka S Inoue T Takenaka M Hasegawa H Yoshida H Ordering cylindrical microdomains for binary blends of block copolymers with graphoepitaxy Macromol. Rapid Commun 2007 28 2137 2144

[128] Cheng JY Mayes AM Ross CA Nanostructure engineering by templated self-assembly of block copolymers Nat. Mater 2004 3 823 828 15467725 10.1038/nmat1211

[129] Bosworth JK Paik MY Ruiz R Schwartz EL Huang JQ Ko AW Smilgies DM Black CT Ober CK Control of self-assembly of lithographically patternable block copolymer films ACS Nano 2008 2 1396 1402 19206307 10.1021/nn8001505

[130] Bita I Yang JKW Jung YS Ross CA Thomas EL Berggren KK Graphoepitaxy of self-assembled block copolymers on two-dimensional periodic patterned templates Science 2008 321 939 943 18703736 10.1126/science.1159352

[131] Rudenko T Kilchytska V Collaert N Nazarov AN Jurczak M Flandre D Electrical characterization and special properties of FINFET structures Nanoscaled Semiconductor-On-Insulator Structures and Devices1st ed Springer Verlag Berlin, Germany 2007

[132] Black CT Ruiz Z Bretya G Cheng JY Colburn ME Guarini KW Kim HC Zhang Y Polymer self assembly in semiconductor microelectronics IBM J. Res. Dev 2007 51 605 633

[133] Segalman RA Schaefer KE Fredrickson GH Kramer EJ Mogonov S Topographic templating of islands and holes in highly asymmetric block copolymer films Macromolecules 2003 36 4498 4506

[134] Park SM Stoykovich MP Ruiz R Zhang Y Black CT Nealey PF Directed assembly of lamellae-forming block copolymers by using chemically and topographically patterned substrates Adv. Mater 2007 19 607 611

[135] Ruiz R Sandstrom RL Black CT Induced orientational order in symmetric diblock copolymer thin films Adv. Mater 2007 19 587 591

[136] Fitzgerald TG Borsetto F O’Callaghan JM Kosmala B Shaw MT Holmes JD Morris MA Polymer nanostructures in sub-micron lithographically defined channels: Film-thickness effects on structural alignment of a small feature size polystyrene-polyisoprene-polystyrene block copolymer Soft Matter 2007 2 916 921 10.1039/b702125a 32900087

[137] Scheel HJ Fukuda T Crystal Growth Technology1st ed John Wiley & Sons, Inc London, UK 2003

[138] Hammond MR Cochran E Fredrickson GH Kramer EJ Temperature dependence of order, disorder, and defects in laterally confined diblock copolymer cylinder monolayers Macromolecules 2005 38 6575 6585

[139] Chastek TQ Lodge TP Twinning and growth kinetics of lamellar grains in a diblock copolymer solution J. Polym. Sci., Part B: Polym. Phys 2005 44 405 412

[140] Harrison C Angelescu DE Trawick M Zhengdong C Huse DA Chaikin PM Vega DA Sebastian JM Register RA Adamson DH Pattern coarsening in a 2D hexagonal system Europhys. Lett 2004 67 800 806

[141] Segalman RA Hexemer A Kramer EJ Edge effects on the order and freezing of a 2D Array of block copolymer spheres Phys Rev Letts 2003 91 196101/1-4 14611589 10.1103/PhysRevLett.91.196101

[142] Hahm J Lopes WA Jaeger HM Sibener SJ Defect evolution in ultrathin films of polystyrene-block-polymethylmethacrylate diblock copolymers observed by atomic force microscopy J. Chem. Phys 1998 109 10111 10114

[143] Hahm J Sibener SJ Time-resolved atomic force microscopy imaging studies of asymmetric PS-*b*-PMMA ultrathin films: Dislocation and disclination transformations, defect mobility, and evolution of nanoscale morphology J. Chem. Phys 2001 114 4730 4740

[144] Tsarkova L Horvat A Krausch G Zvelindovsky AV Sevink GJA Magerle R Defect evolution in block copolymer thin films via temporal phase transitions Langmuir 2006 22 8089 8095 16952246 10.1021/la0613530

[145] Stoykovich MP Kang H Daoulas KC Liu G Liu CC de Pablo JJ Müller M Nealey PF Directed self-assembly of block copolymers for nanolithography: Fabrication of isolated features and essential integrated circuit geometries ACS Nano 2007 1 168 175 19206647 10.1021/nn700164p

[146] Roerdink M Hempenius MA Gunst U Arlinghaus HF Vancso GJ Substrate wetting and topographically induced ordering of amorphous PI-*b*-PFS block-copolymer domains Small 2007 3 1415 1423 17615588 10.1002/smll.200700044

[147] Welander AM Nealey PF Cao H Bristol R Impact of trench width roughness on the graphoepitaxial assembly of block copolymers J. Vac. Sci. Tech. B 2008 26 2484 2488

[148] Park SM Craig GSW La YK Nealey PF Morphological reconstruction and ordering in films of sphere-forming block copolymers on striped chemically patterned surfaces Macromolecules 2008 41 9124 9129

[149] Huck WTS Effects of nanoconfinement on the morphology and reactivity of organic materials Chem. Comm 2005 41 4143 4148 16100584 10.1039/b502849n

[150] Black CT Self-aligned self assembly of multi-nanowire silicon field effect transistors Appl. Phys. Lett 2005 87 163116

[151] Ruiz R Ruiz N Sandstrom RL Black CT Local defectivity control of 2D self-assembled block copolymer patterns Adv. Mater 2007 19 2157 2162

